# Magnetic Resonance Imaging of Atherosclerotic Plaque at Clinically Relevant Field Strengths (1T) by Targeting the Integrin α4β1

**DOI:** 10.1038/s41598-018-21893-x

**Published:** 2018-02-27

**Authors:** Darren G. Woodside, Eric A. Tanifum, Ketan B. Ghaghada, Ronald J. Biediger, Amy R. Caivano, Zbigniew A. Starosolski, Sayadeth Khounlo, Saakshi Bhayana, Shahrzad Abbasi, John W. Craft, David S. Maxwell, Chandreshkumar Patel, Igor V. Stupin, Deenadayalan Bakthavatsalam, Robert V. Market, James T. Willerson, Richard A. F. Dixon, Peter Vanderslice, Ananth V. Annapragada

**Affiliations:** 1 Department of Molecular Cardiology, Texas Heart Institute, 6770 Bertner Avenue, Houston, Texas 77030 USA; 2 Department of Pediatric Radiology, Texas Children’s Hospital, 6621 Fannin Street, Houston, Texas 77030 USA; 3 Department of Biology and Chemistry, University of Houston, 4800 Calhoun Road, Houston, Texas 77004 USA; 4 Department of Experimental Therapeutics, University of Texas MD Anderson Cancer Center, 1515 Holcombe Boulevard, Houston, Texas 77030 USA; 5 Division of Cardiology Research, Texas Heart Institute, 6770 Bertner Avenue, Houston, Texas 77030 USA; 6 Present Address: Department of Institutional Analytics and Informatics, University of Texas MD Anderson Cancer Center, Houston, Texas USA

## Abstract

Inflammation drives the degradation of atherosclerotic plaque, yet there are no non-invasive techniques available for imaging overall inflammation in atherosclerotic plaques, especially in the coronary arteries. To address this, we have developed a clinically relevant system to image overall inflammatory cell burden in plaque. Here, we describe a targeted contrast agent (THI0567-targeted liposomal-Gd) that is suitable for magnetic resonance (MR) imaging and binds with high affinity and selectivity to the integrin α4β1(very late antigen-4, VLA-4), a key integrin involved in recruiting inflammatory cells to atherosclerotic plaques. This liposomal contrast agent has a high T1 relaxivity (~2 × 10^5^ mM^−1^s^−1^ on a particle basis) resulting in the ability to image liposomes at a clinically relevant MR field strength. We were able to visualize atherosclerotic plaques in various regions of the aorta in atherosclerosis-prone ApoE^−/−^ mice on a 1 Tesla small animal MRI scanner. These enhanced signals corresponded to the accumulation of monocyte/macrophages in the subendothelial layer of atherosclerotic plaques *in vivo*, whereas non-targeted liposomal nanoparticles did not demonstrate comparable signal enhancement. An inflammatory cell-targeted method that has the specificity and sensitivity to measure the inflammatory burden of a plaque could be used to noninvasively identify patients at risk of an acute ischemic event.

## Introduction

Global mortality rates identify ischemic heart disease as a leading cause of death^1^. Thrombosis resulting from degradation of atherosclerotic plaques, either through plaque rupture or superficial erosion, is the cause of a majority of acute coronary ischemic events in humans^2–5^. Although therapeutic targeting of risk factors for atherosclerosis, such as high blood pressure and cholesterol levels, has reduced mortality rates, these same risk factors are poor indicators of acute or recurrent events^6^. Despite our improved understanding of the physiology of atherosclerotic plaque development and progression, no clinically relevant imaging tools are available to identify patients who are at high risk of having an ischemic coronary event.

Atherosclerosis is an inflammatory disease^7^ that begins with the subendothelial accumulation of lipoproteins in small- to medium-sized arteries^8^. This accumulation results in activation of endothelial cells, which involves upregulating the expression of adhesion molecules, including vascular cell adhesion molecule-1(VCAM-1). By binding integrin receptors, such as α4β1 on circulating leukocytes, these adhesion molecules facilitate the recruitment and accumulation of leukocytes at inflammatory sites^9–11^. Monocytes/macrophages and lymphocytes make up about half of the cellular components of atherosclerotic plaques^12^; neutrophils are present in lower numbers^13^. Because these cell types are found at the site of culprit lesions and contribute to the weakening of plaques, they are considered critical markers of plaques that are vulnerable to rupture^12,14–17^ or erosion^18,19^.

Current clinical diagnostic techniques cannot be used to assess the complete inflammatory cell burden in atherosclerotic plaques. Coronary angiography, although the standard technique for imaging arterial anatomy, and contrast-enhanced computed tomography cannot delineate the cellular and molecular composition of plaques^20,21^. ^18^F-fluorodeoxyglucose (^18^F-FDG)–based positron emission tomography (PET) imaging can be used to measure inflammation^22^ but lacks specificity for inflammatory cells as it is taken up by metabolically active cells in the myocardium^21^. Other limitations of ^18^F-FDG-PET include radiation doses that limit longitudinal studies and restricted geographic availability of PET tracers. ^18^F-modified polyglucose nanoparticles (^18^F-Macroflor), or the PET tracer ^68^Ga-DOTATATE (which only binds a subset of macrophage that express the somatostatin receptor subtype-2 (SST_2_), allow for quantification of macrophages but not lymphocytes or neutrophils^23,24^. MRI with conventional gadolinium (Gd) contrast agents also lacks specificity for detecting inflammatory cells at atherosclerotic sites. Molecular imaging agents that target cell surface receptors expressed on immune cells or molecules expressed specifically within atherosclerotic plaque have been evaluated in preclinical settings^25–31^. Peptides^32–34^, monoclonal antibodies^35,36^, single-chain antibodies^37^, and nanobodies^38^ have been used in these imaging constructs; however, clinical translation of these agents is hampered by concerns of *in-vivo* stability, safety-toxicity, and clinical manufacturing.

Approaches targeting integrin receptors have been investigated for imaging inflammatory plaques. However, the majority of integrin-targeted constructs under development for molecular imaging of inflammation in atherosclerosis rely on RGD (Arg-Gly-Asp) peptides and their variants, despite the fact that the main integrins responsible for inflammatory cell recruitment into atherosclerotic plaque are non-RGD binding integrins. Integrin α4β1 is a non-RGD binding integrin expressed on monocytes/macrophages, lymphocytes^39^, and neutrophils^40^. Integrin α4β1 binds the counter-receptor VCAM-1 on activated endothelial cells; this binding recruits monocytes/macrophages and T cells to atherosclerotic plaques^9–11^. Integrin α4β1 is a clinically validated drug target^41^ for which high-affinity, small-molecule antagonists have been identified^42,43^. As such, integrin α4β1 could facilitate assessment of the overall immune cell burden of atherosclerotic plaques and, therefore, is an attractive candidate for the development and clinical translation of imaging agents targeting inflamed plaques.

The aim of this work was to develop a targeted MR imaging agent that would enable imaging of inflammatory cell burden in atherosclerotic plaques using a system that would have broad clinical applicability. To this end, we have incorporated a novel non-peptidic small molecule integrin α4β1 antagonist into a liposomal Gd contrast agent. Integrin α4β1 directs inflammatory cells to atherosclerotic plaques^9–11^. Liposomes are considered safe in humans, and their pharmacokinetics, organ distribution, and toxicities are well understood^44^. The targeted liposomal Gd contrast agents used in our study enable imaging of relevant cell types within atherosclerotic plaques at clinically relevant field strengths. These unique features of our approach should facilitate translation to clinical development.

## Results

### Modifications of integrin α4β1 antagonists

**Figure 1:**
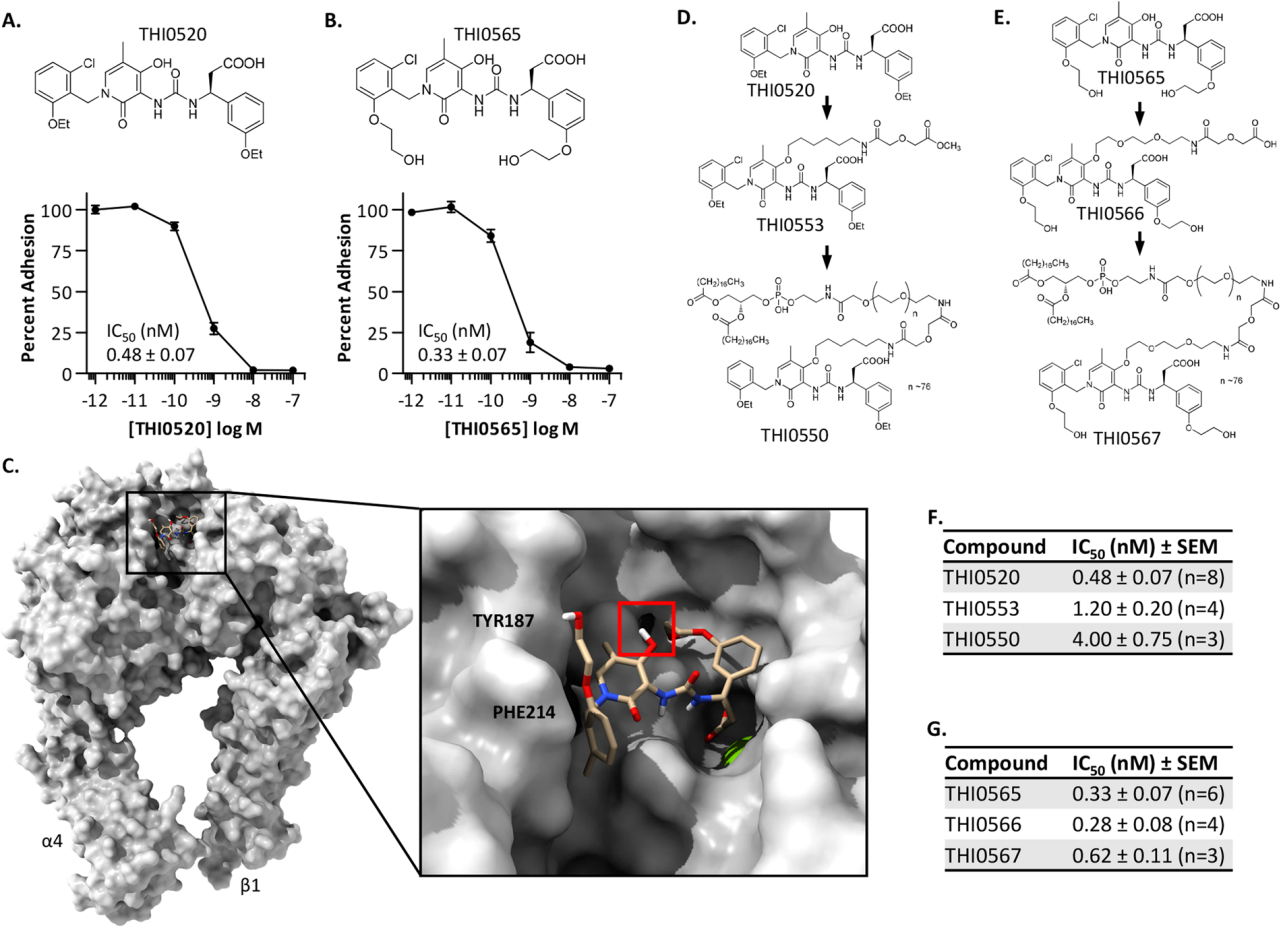
Modification of small molecule antagonists of the integrin α4β1. (**A**,**B**) Structure and activity of the small molecule integrin antagonists. Studies showing α4β1-expressing-K562 cell adhesion to plastic-immobilized vascular cell adhesion molecule-1 (VCAM-1). An average “Percent Adhesion” from at least 6 independent experiments is shown (average ± SEM). (**C**) Molecular Dynamics simulation of THI0565 binding into the integrin α4β1 ectodomain. THI0565 is anchored by the carboxylic acid coordination of the β1 MIDAS Mg^++^ ion (green sphere, expanded 2x for visualization) and the 2-hydroxyethoxyphenyl group hydrophobic interactions with α4 residues PHE214 and TYR187. The pyridone hydroxyl appears readily available for modification (red square). (**D**,**E**) Modifications of THI0520 and THI0565 to generate targeting conjugates for liposome formulation. (**F**,**G**) Inhibitory activity (IC_50_) of modified compounds as determined in α4β1-K562 cell adhesion assays to VCAM-1.

We have developed multiple structural classes of small molecule antagonists of the integrin α4β1^45–48^. THI0520 and THI0565 (Fig. 1A,B) are compounds belonging to a non-peptidic structural class and are potent antagonists of the integrin α4β1 (IC_50_, 0.48 ± 0.07 nM [n = 8] and 0.33 ± 0.07 nM [n = 6], respectively). Conversely, they demonstrated decreased affinity against integrin heterodimers that are related to integrin α4β1, namely α4β7 and α9β1, and showed no activity against other integrins not related to α4β1 (Supplemental Table S1). Previous structure-activity relationship (SAR) analyses identified the central pyridone ring region of these compounds as an area that could be modified with little effect on antagonist affinity. Molecular Dynamics simulations of these antagonists within the ligand-binding domain of integrin α4β1 correlated well with previous SAR analyses in that the major binding determinants of THI0565 included the carboxylic acid coordinating the divalent cation in the β1 subunit metal ion-dependent adhesion site (MIDAS) and 2-hydroxyethoxyphenyl hydrophobic interactions with the α4 subunit PHE214 and TYR187 (Fig. 1C). The hydroxyl group of the central pyridone ring appeared to be in a region that could be modified and would not affect antagonist affinity (Fig. 1C, right panel). On the basis of SAR and modelling results, we modified THI0520 and THI0565 at the central pyridone ring hydroxyl group with linkers and functional groups (Fig. 1D,E) to incorporate these antagonists into liposomes for use as targeting ligands (see Compound Synthesis in Supplement). Modifying these compounds with a 12- or 14-atom chain that included a terminal carboxylic acid/ester, then further with DSPE-PEG_3400_ (to incorporate into liposomes), resulted in only a 2–10-fold decrease in apparent affinity. Specifically, DSPE-PEG_3400_ modification of THI0520 (to generate compound THI0550) decreased antagonist affinity almost 10-fold, from 0.48 ± 0.20 nM (n = 8) to 4.0 ± 1.3 nM (n = 3, Fig. 1F), whereas similar modification of THI0565 (to create compound THI0567) decreased antagonist affinity only from 0.33 ± 0.16 nM (n = 6) to 0.62 ± 0.19 nM (n = 3) (Fig. 1G). THI0567 remained the most potent compound after modification and was used to prepare the targeted liposomal formulations.

### Generation of integrin α4β1 targeted liposomes

**Figure 2:**
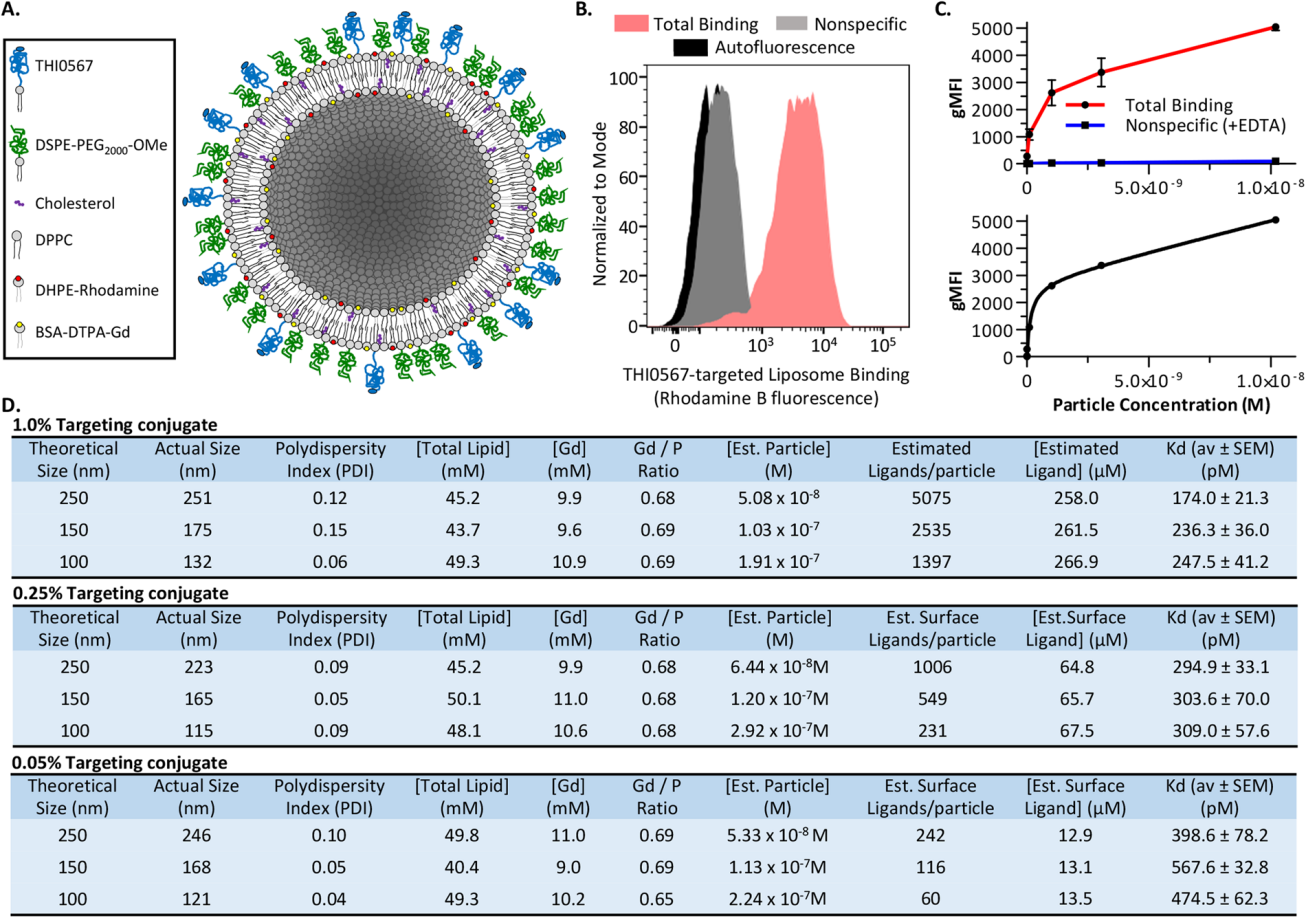
Integrin α4β1-targeted liposomes. (**A**) Schematic diagram of liposome formulations. (**B**) Flow cytometric analysis of THI0567-targeted liposome (250 nm, 1.0% targeting conjugate, 3 × 10^−9^ M) binding to Jurkat cells. Background nonspecific binding was measured in the presence of EDTA (20 mM). Three separate experiments were overlayed into one histogram. Nonspecific binding was determined in the presence of EDTA (20 mM) (**C**) Dose-dependent binding of THI0567-targeted liposomes (250 nm, 1.0% targeting conjugate) to Jurkat cells. Curve fitting was performed with GraphPad Prizm using a one-site binding model of total and nonspecific binding (lower graph). Liposome binding is expressed as the geometric mean fluorescence intensity (gMFI) of rhodamine B fluorescence. Data are expressed as mean ± SEM (n = 3 independent experiments). (**D**) Effects of particle size and targeting conjugate densities on liposome performance. Average Kds were calculated from 3 separate experiments for each liposome formulation. Abbreviations: DPPC-Dipalmitoyl phosphatidylcholine; DSPE-PEG_2000_-OMe - Distearoyl phosphoethanolamine- methoxy polyethylene glycol_2000_; THI0567 - Distearoyl phosphoethanolamine-methoxy-polyethylene glycol_3400_ –linker-THI0565; DHPE-Rhodamine - Dihexadecanoyl phosphoethanolamine (lissamine rhodamine B); DTPA-BSA-Gd - Diethylene triamine pentaacetic acid-bis(stearylamide) (gadolinium).

Although unmodified liposomal nanocarriers are internalized by phagocytic cells to a limited extent, attaching targeting agents to these carriers has dramatically increased the efficiency and specificity of cellular uptake due to receptor-mediated endocytosis^49,50^. We generated a matrix of liposome formulations (for liposome constituents, see Supplementary Table S2) to test the effects of liposome size (between 100–250 nm) and surface density of the targeting ligand (0.05–1.0% total lipid concentration) on the efficiency of liposome binding to integrin α4β1–expressing cells. Figure 2A shows a schematic of the liposome variants. To develop the MRI contrast agent, we incorporated Gd into the liposome bilayer by using methods described previously^51^. Rhodamine-labeled liposomal formulations were used for fluorescence-based *in-vitro* and *in-vivo* binding analyses. Flow cytometry-based binding assays were performed by using the integrin α4β1–expressing human Jurkat T cell line^52^ (Fig. 2B). Strong binding of THI0567-targeted liposomes (red histogram) was detected over cellular autofluorescence. Nonspecific binding was measured in the presence of EDTA, which chelates the divalent cation Mg^2+^ from the β1 chain MIDAS site, inactivating the integrin^53^. Nonspecific binding was minimal (Fig. 2B). Dose-response assays were performed with and without EDTA to generate total and nonspecific binding curves. Targeted liposome binding was dose dependent and saturable (Fig. 2C). Binding data were fit by using a one-site binding model for all liposome variants in the matrix (Supplementary Fig. S1). Liposome physical characteristics, chemical composition, and binding activities are summarized in Fig. 2D. Binding affinity directly correlated with THI0567 (targeting ligand) concentration and liposome size. Larger liposome size (>200 nm) results in a shorter blood circulation half-life because of rapid clearance by cells of the reticuloendothelial system, and the larger size presents challenges in clinical translation due to issues with preparation of sterile formulation. Thus, we selected liposomes with incorporation of 1% targeting ligand and an average diameter of 150–175 nm for further study.

### Binding specificity and selectivity of α4β1-targeted liposomes

**Figure 3:**
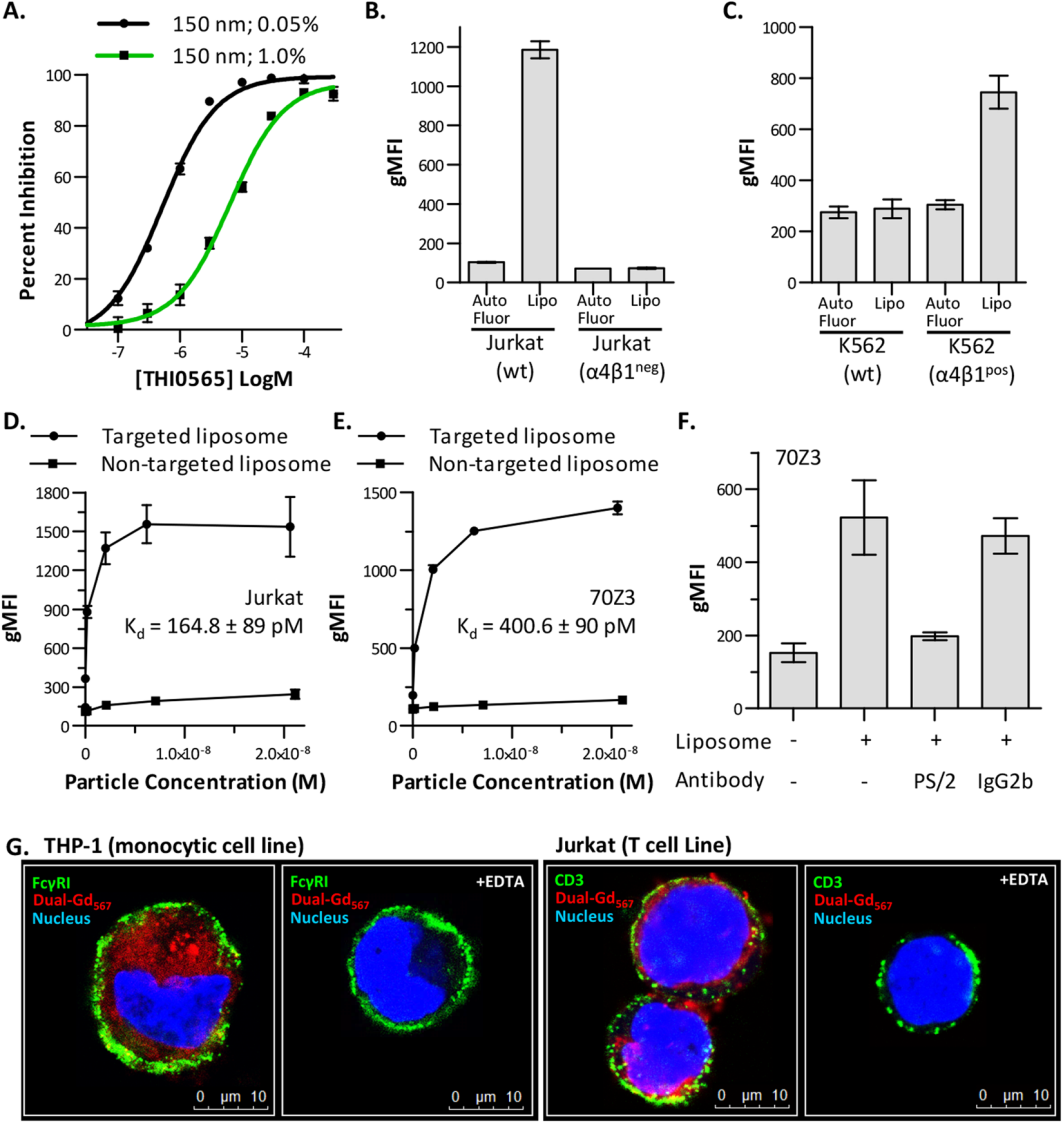
α4β1-specific binding of THI0567-targeted liposomes. (**A**) THI0565 competition with THI0567-targeted liposome (150 nm; 0.05% or 1.0% targeting conjugate; used at a particle concentration of 2 × 10^−10^ M). Average %inhibition ± SEM (n = 3 experiments). (**B)** Binding of THI0567-targeted liposome (2 × 10^−10^ M) to wildtype Jurkat cells and α4β1^neg^ Jurkat cells (**C)** Binding of THI0567-targeted liposome (2 × 10^−10^ M) to wildtype K562 cells and a K562 cells stably expressing integrin α4β1 (α4β1^pos^). (**D**,**E)** THI0567-targeted liposome (150 nm; 1.0% targeting conjugate) and non-targeted liposome (150 nm; 0% targeting conjugate) binding to Jurkat and murine 70Z3 cells. In (**B–E)**, average gMFI ± SEM (n = 3 experiments). (**F)** THI0567-targeted liposome binding to murine 70Z3 cells in the presence of anti-α4 mAb PS/2 or IgG2b isotype control (average gMFI ± SEM [n = 4 experiments]). (**G)** THI0567-targeted (Dual-Gd_567_) liposomes (2 × 10^−10^ M) were incubated with THP-1 or Jurkat T cells (1 h at RT, ± 20 mM EDTA), followed by counter-staining for FcγRI (green, THP-1) and CD3 (green, Jurkat). Blue; Hoechst 33342.

THI0565, the α4β1 integrin antagonist modified to generate THI0567 (Fig. 1F), was used to demonstrate the specificity of THI0567-targeted liposome binding to Jurkat T cells. In competition experiments, THI0565 inhibited THI0567-targeted liposome binding to Jurkat cells in a dose-dependent manner. The half-maximal inhibitory concentrations of THI0565 were 6.4 × 10^−6^ M when tested against targeted liposome containing 1.0% targeted ligand and 5.2 × 10^−7^ M when tested against targeted liposome containing 0.05% targeted ligand (Fig. 3A). Decreasing the ligand concentration of the liposome by 20-fold resulted in a corresponding 12-fold decrease in the IC_50_ of THI0565; this further supports the idea that binding of liposomes to Jurkat cells is dependent on the targeting ligand. Binding specificity was also verified by comparing binding of THI0567-targeted liposomes to wildtype Jurkat cells or to Jurkat cells treated with mutagen and selected for loss of integrin α4β1 expression^54^ (Supplementary Fig. S2). There was no specific binding to α4β1-deficient Jurkat cells (Fig. 3B) or to the erythroleukemic cell line K562 (Fig. 3C), which has no detectable levels of α4β1 (Supplementary Fig. S2). However, when the α4 subunit was stably expressed in K562 cells (Supplementary Fig. S2), THI0567-targeted liposomes bound (Fig. 3C). Non-targeted liposomes were generated and binding assays were performed to compare them against THI0567-targeted liposomes. Non-targeted liposome binding to Jurkat cells was low (Fig. 3D). THI0567-targeted liposomes specifically bound α4β1-expressing murine 70Z3 cells with similar affinity to human cells, demonstrating cross-species binding (Fig. 3E). Similar binding was also observed for α4β1-expressing rat, rabbit, and canine cells (Supplementary Table S2). Binding was dependent on integrin α4β1 because function-blocking antibodies to this integrin prevented the interaction (Fig. 3F). Confocal analysis of THI0567-targeted liposome binding to monocytic cells (THP-1) and T cells (Jurkat) indicated liposome internalization (Fig. 3G), which was verified with a 3D reconstruction from confocal Z-stack images of internalized liposome in Jurkat T cells (Supplementary Movie S1).

### *In vivo* binding of targeted liposomes

**Figure 4:**
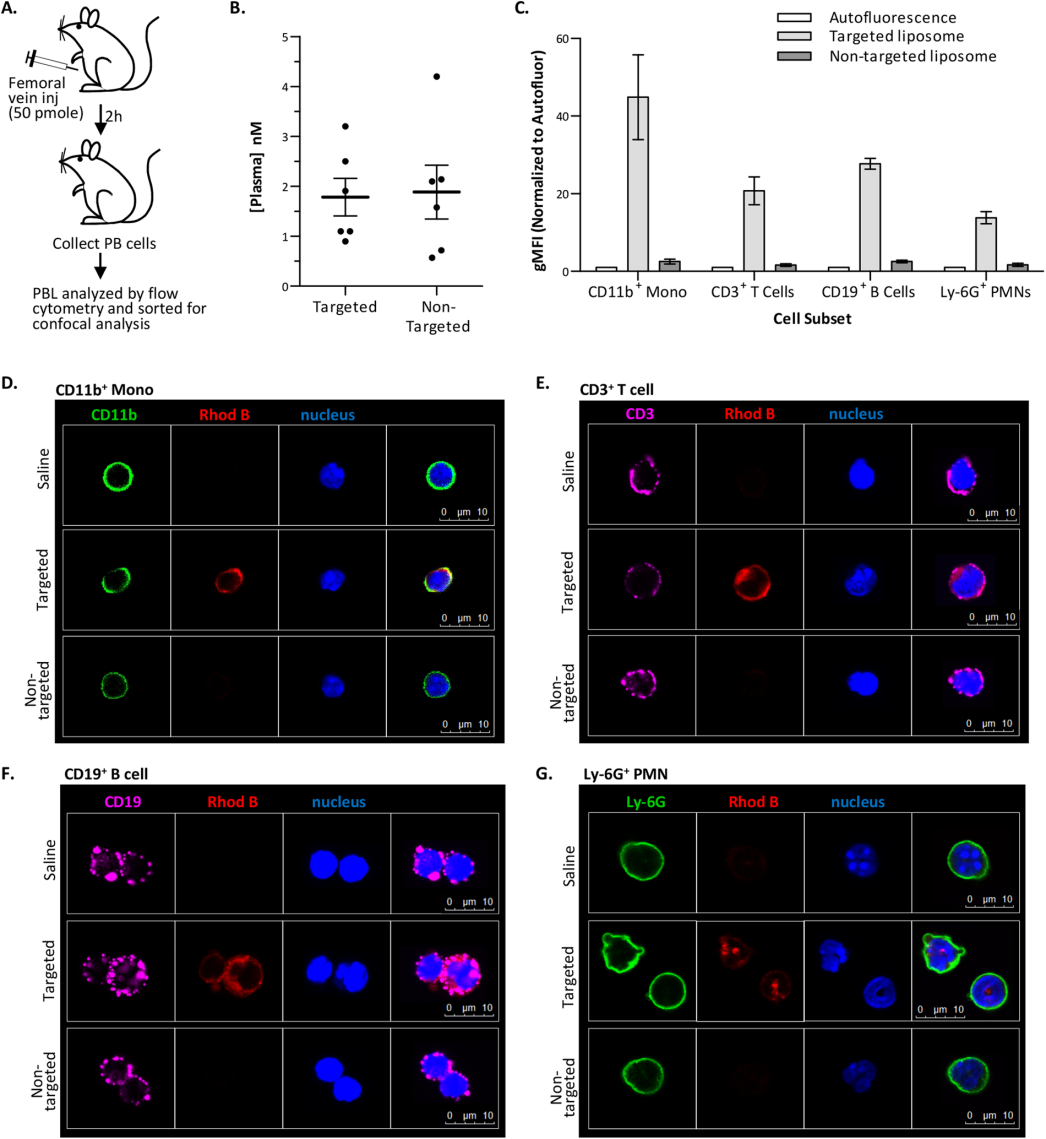
*In vivo* binding of targeted liposomes. (**A**) Schematic of experimental design. (**B)** Rhodamine B levels in the plasma (2 h after dosing). (**C**) Rhodamine B fluorescence in sorted peripheral blood (PB) cells used for confocal analysis. Average gMFI ± SEM from 3 experiments (normalized for cellular autofluorescence). (**D–G**) Confocal imaging of liposome binding to PB cells. After cytometry and sorting, confocal microscopy was performed for CD11b (FITC) (**D**), CD3 (Cy5) (**E**), CD19 (Cy5) (**F**), Ly-6G (FITC) (**G**), Rhodamine B (to identify liposomes), and Hoechst 33342 (nucleus). Identical confocal acquisition settings were used for all images. For all experiments, targeted liposome = THI0567-targeted liposomal-Gd (150 nm; 1.0% targeting conjugate); non-targeted = liposomal-Gd (150 nm; 0% targeting conjugate). Abbreviations: PBL (peripheral blood lymphocytes); gMFI (geometric mean fluorescence intensity); SEM (standard error of the mean); CD (cluster of differentiation); PMN (polymorphonuclear).

Before performing *in vivo* experiments, we determined that plasma did not adversely affect specific binding of targeted liposomes to Jurkat cells (Supplementary Fig. S3). Then, we analyzed the binding of targeted liposomes to subsets of peripheral blood cells *in vivo*. For these studies, healthy C57BL/6 mice were injected (via the femoral vein) with saline vehicle, THI0567-targeted liposomes, or non-targeted liposomes (Fig. 4A). Peripheral blood was collected 2 h after injection. Plasma was stored to measure liposome concentrations (Fig. 4B), and cells were stained for the indicated markers. Quantification by flow cytometry demonstrated specific uptake of THI0567-targeted liposomes in CD11b^+^ mononuclear cells, CD3^+^ T cells, CD19^+^ B cells, and Ly-6G^+^ polymorphonuclear leukocytes but negligible uptake of non-targeted liposome in any of these populations (Fig. 4C). The cells were sorted and fixed onto glass slides for confocal microscopy. Imaging of Rhodamine B fluorescence corroborated the flow cytometric results. Rhodamine B fluorescence was observed in peripheral blood CD11b^+^ mononuclear cells and CD3^+^ T cells isolated from mice injected with THI0567-targeted liposome (Fig. 4D,E). However, in mice injected with non-targeted liposomes, Rhodamine fluorescence was similar to saline-treated animals (Fig. 4D,E). Similar results were found when CD19^+^ B cells and Ly-6G^+^ polymorphonuclear leukocytes were examined (Fig. 4F,G). The lack of non-targeted liposome uptake was not due to a lower concentration of this agent in plasma because we found no significant difference between THI0567-targeted and non-targeted liposome concentrations in the plasma at the 2 h time point (Fig. 4B).

### Liposome uptake in plaques of ApoE^−/−^ mice

**Figure 5:**
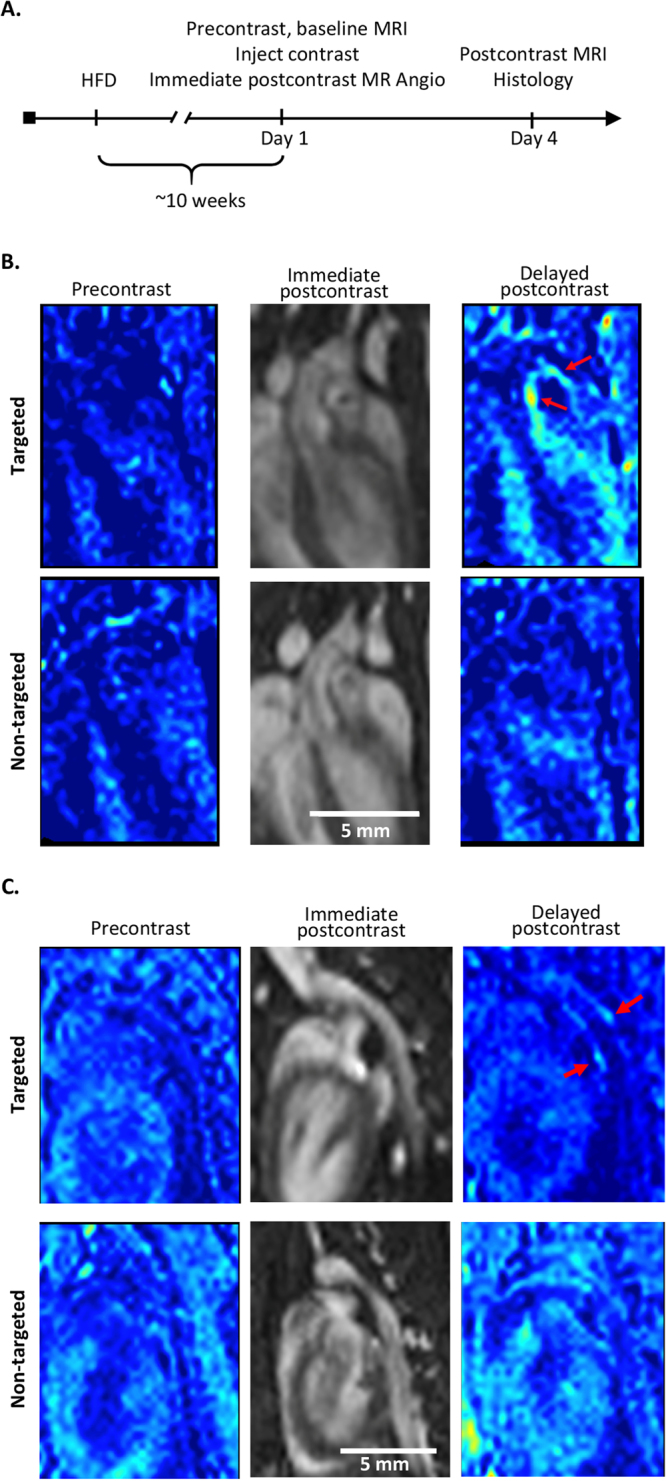
*In vivo* MRI of atherosclerotic plaques. (**A**) Timeline for MRI studies. ApoE^−/−^ mice (12–14 weeks old) were fed a high-fat diet (HFD) for ~10 weeks before initiating the start of imaging. MRI was performed at 3 time points: (1) precontrast, (2) immediate postcontrast (within 1 hour of contrast administration to acquire an angiogram of the aorta), and (3) delayed postcontrast at 72 h after contrast administration. (**B**) Representative coronal T1-weighted pseudo-colored MR images of the aortic arch from a THI0567-targeted liposomal-Gd treated mouse (top row) and a non-targeted liposomal-Gd-treated mouse (bottom row) at baseline, immediate postcontrast, and delayed postcontrast. Signal enhancement within the aortic arch wall in a THI0567-targeted liposomal-Gd-treated mouse (red arrow) is shown. (**C**) Representative coronal T1-weighted pseudo-colored MR images of the descending aorta from a THI0567-targeted liposomal-Gd-treated mouse (top row) and a non-targeted liposomal-Gd treated-mouse (bottom row). Signal enhancement is seen in the aortic wall in the THI0567-targeted liposomal-Gd treated mouse (red arrows).

**Figure 6:**
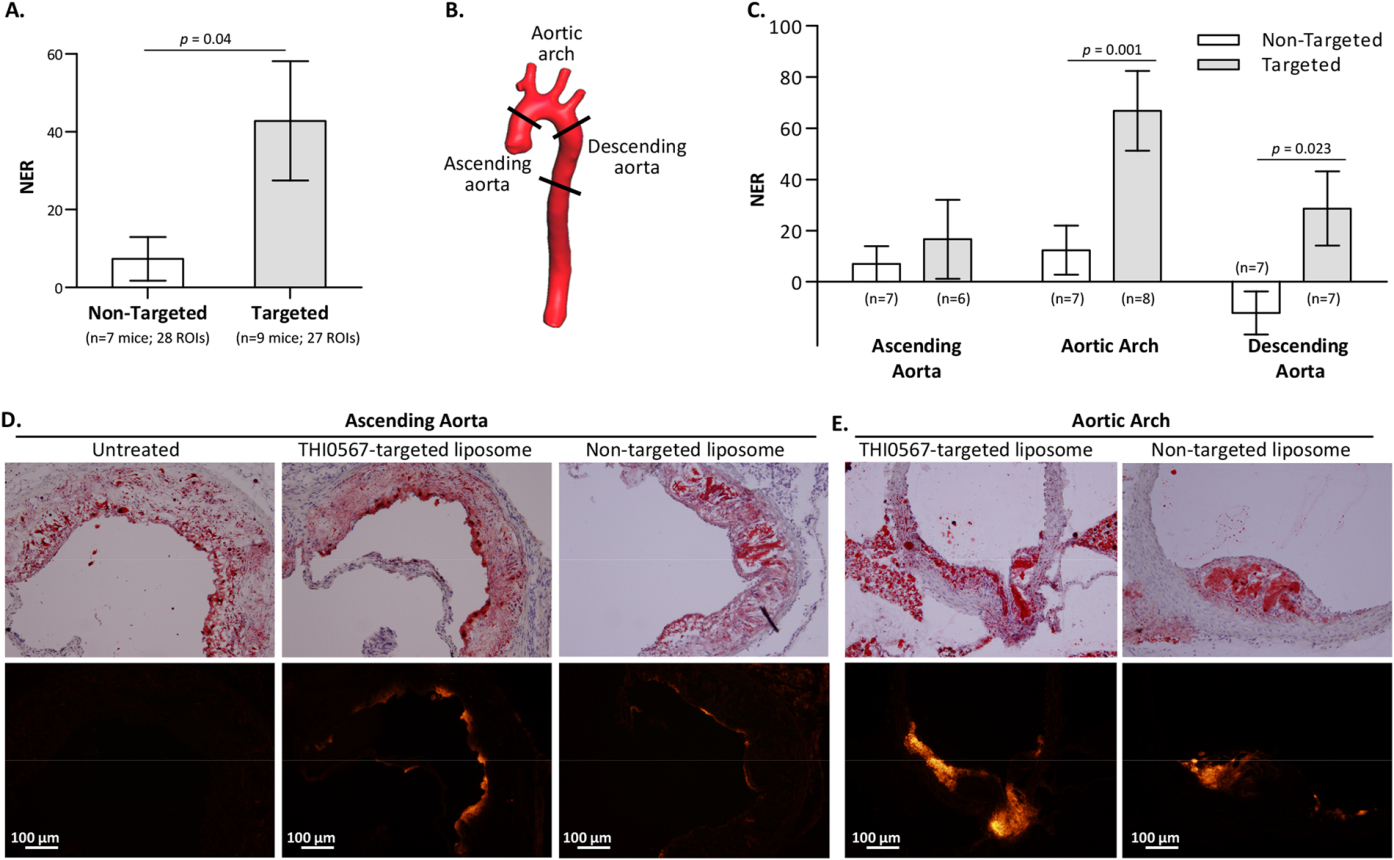
Quantitative analysis of MRI signal enhancement in atherosclerotic plaques. (**A**) Comparison of the normalized enhancement ratio (NER) of atherosclerotic plaques in non-targeted (n = 7) and THI0567-targeted groups of mice (n = 9). Data are presented as average ± SEM. (**B**) Volume-rendered 3D image of a mouse aorta showing the different aortic segments examined. (**C**) Comparison of the NER in atherosclerotic plaques in non-targeted (n = 7) and targeted groups of mice (n = 6–8) for different segments of the aorta. Data are presented as average ± SEM. (**D**,**E**) Representative sections of oil red staining (upper panels) and Rhodamine B fluorescence (lower panels) from the ascending aorta (**D**) and aortic arch (**E**) regions of untreated, THI0567-targeted liposomal-Gd, or non-targeted liposomal-Gd injected mice.

We examined THI0567-targeted liposome uptake in atherosclerotic plaque in mice. ApoE^−/−^ mice fed a pro-atherogenic diet develop inflammatory plaques predominantly in the aortic root and arch regions and at branch points of the arterial tree^55^. Inflammatory cell recruitment into these plaques is largely due to the integrin α4β1/VCAM-1 adhesion axis^9–11^. For our MRI studies, ApoE^−/−^ mice (12–14 weeks old) were fed a high-fat diet for approximately 10 weeks. Then, the mice were intravenously injected with THI0567-targeted liposomes (0.1 mmol Gd/kg) or non-targeted control liposomes (0.1 mmol Gd/kg). *In vivo* MR imaging was performed on a 1 T permanent magnet using a T1-weighted (T1w) 3D gradient-recalled echo (GRE) sequence. All mice underwent precontrast T1w imaging followed by administration of liposomal-Gd contrast agent (THI0567-targeted or non-targeted) and an immediate postcontrast scan. To ensure clearance of the contrast agent from the circulation, we acquired delayed postcontrast scans 72 hours after administering the liposomal contrast agent (Fig. 5A). The THI0567-targeted liposomal-Gd agent showed higher aortic wall signal enhancement on T1w images at multiple locations along the aorta (Fig. 5B,C); the non-targeted agent did not show comparable signal enhancement. To quantitate signal enhancement in MR images, we calculated normalized enhancement ratios (NER) using previously described methods^56^. The overall NER was significantly higher in mice administered the THI0567-targeted liposomal-Gd agent than in those given the non-targeted liposomal-Gd (42.8 ± 15.3 vs. 7.4 ± 5.6, p = 0.04) (Fig. 6A). To identify regions with the highest signal enhancement, we determined the NER in multiple aortic segments: the ascending aorta, aortic arch, and descending aorta (Fig. 6B). The NER was significantly higher in targeted liposomal-Gd-treated mice than in mice administered non-targeted liposomal-Gd in the aortic arch (66.8 ± 15.6 vs. 12.3 ± 9.6, p = 0.001) and the descending aorta (28.7 ± 14.6 vs. −12.2 ± 8.4, p = 0.023) (Fig. 6C). In the ascending aorta, although the average NER was higher in the targeted-liposome group, the difference between the two groups did not reach statistical significance. After the delayed postcontrast scans, aortic tissue was harvested and sectioned to examine liposome accumulation patterns. On examination of sections from the ascending aorta and aortic arch, liposome accumulation (as indicated by Rhodamine B fluorescence) was limited to Oil red-positive plaques (Fig. 6D,E). No Rhodamine B fluorescence was observed in plaques from untreated mice (Fig. 6D).

**Figure 7:**
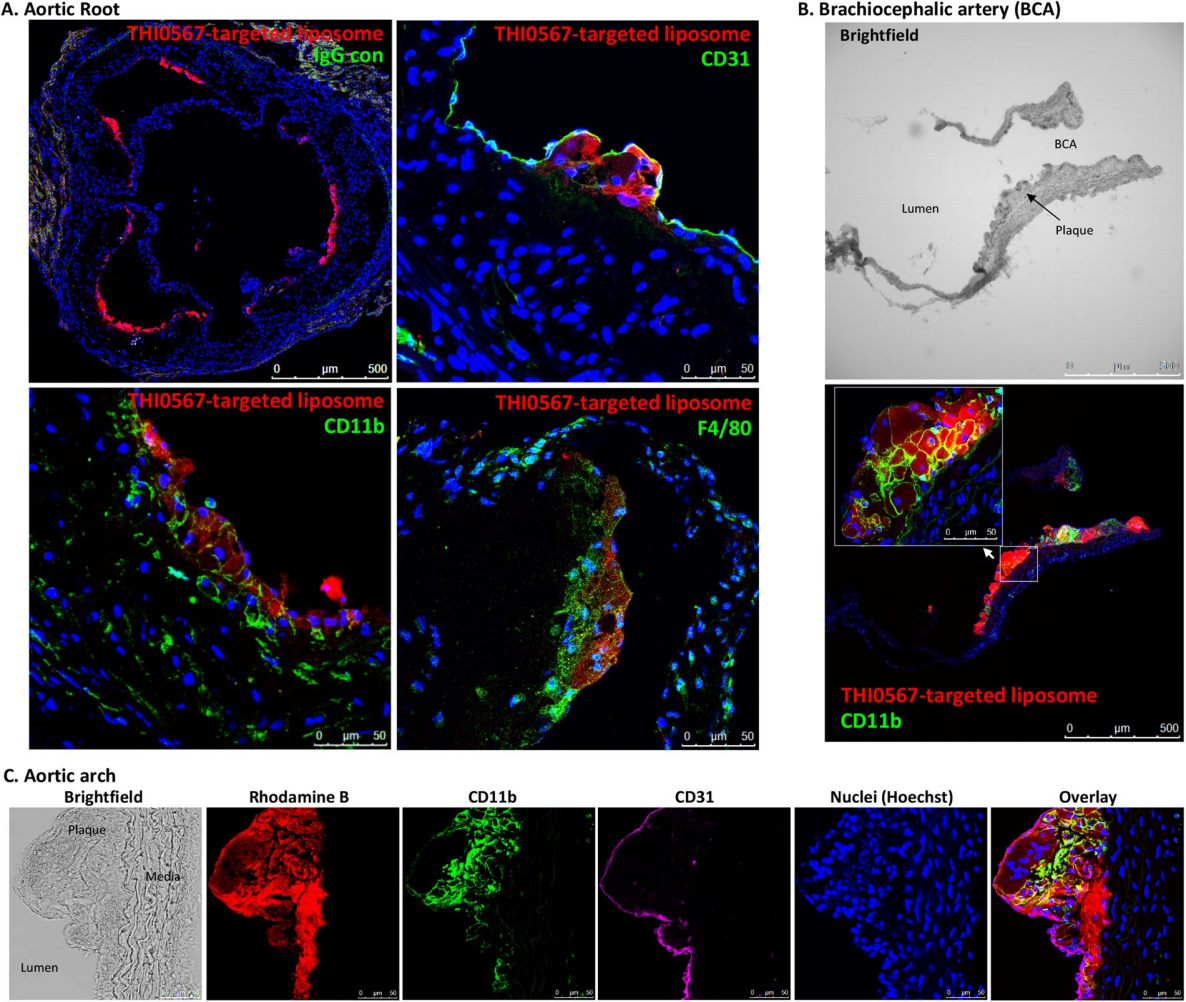
THI0567-targeted liposomal-Gd accumulation in atherosclerotic plaques of ApoE^−/−^ mice. (**A**) Confocal fluorescence imaging of histological sections of the aortic root from mice injected with THI0567-targeted liposomal-Gd (Rhodamine B fluorescence). Control IgG staining is represented in the low magnification (10x) image of the aortic root (upper left panel). Other representative sections were stained with anti-CD31 (upper right), anti-F4/80 (lower right), or anti-CD11b (lower left). Nuclei were stained with Hoechst 33342. (**B**) Partial section through the aortic arch, including the brachiocephalic artery, stained with anti-CD11b and Hoechst 33342. (**C**) Confocal fluorescence imaging of aortic arch sections of THI0567-targeted liposomal-Gd treated animals (representative sections from n = 8. Fluorescence: THI0567-targeted liposomal-Gd (Rhodamine B); CD11b (green); CD31 (purple); nuclei (Hoechst 33342; blue).

Sections of aortic plaques were examined by immunofluorescence to determine if markers associated with cells of the monocytic lineage colocalized with THI0567-targeted liposomes. Rhodamine B fluorescence (liposome) in the aortic root was limited to subendothelial areas within plaques (Fig. 7A, upper right panel). Liposome fluorescence colocalized with both CD11b and F4/80 monocyte/macrophage markers (Fig. 7A, lower panels). Branching arteries, such as the brachiocephalic artery, and the aortic arch region contained dense THI0567-targeted liposome accumulation (Fig. 7B,C, respectively), with liposomes internalized within CD11b^+^ cells in the plaque regions.

## Discussion

No relevant, clinically available noninvasive techniques have been developed to analyze the total inflammatory cell burden in atherosclerotic plaques. Because outcomes-based clinical scores are not accurate in predicting patients who are at high risk of acute ischemic events^57^, there is a need to develop noninvasive techniques that could be widely available and accurately stratify patient risk and possibly guide clinical trials. Here, we have shown that potent small molecule antagonists of the integrin α4β1, a key receptor on inflammatory leukocytes that mediates cell recruitment to atherosclerotic plaques^9–11^, can be modified for use in targeting liposomal-gadolinium contrast agents to α4β1-expressing inflammatory cells. This approach can facilitate molecular MR imaging of inflamed atherosclerotic plaques at clinically relevant MR field strength.

The integrin class of cell surface receptors are attractive targets for imaging probes. They demonstrate tissue-specific expression^58^, can be targeted with small molecule drugs^43,59–61^, and are internalized upon ligand binding^62^. Indeed, several antibody, peptide, and small molecule–based imaging approaches have been described that target mostly integrins αVβ3 for neovascularization^63^ or αIIbβ3 for thrombosis or platelet activation^64^. We have focused on developing liposomal nanoparticles that target the integrin α4β1, a non–RGD-binding integrin, which is expressed on monocytes, lymphocytes, and neutrophils at levels of up to 10^5^ receptors per cell^65^. Integrin α4β1 binds the counter-receptor VCAM-1 on activated endothelial cells. This interaction is critical in several inflammatory disease models^43,48,61,66^ and is responsible for recruiting leukocytes to atherosclerotic plaques^9–11,67^. Here, we developed small molecule antagonists of α4β1 that are potent (IC_50_ ranges between 330 × 10^−12^ to 480 × 10^−12^ M) and selective for α4β1. No activity was seen against integrins α1β1, α2β1, α5β1, αLβ2, αVβ3, and αIIbβ3. These antagonists have a similar structure to those that have completed phase I clinical testing^43^. Using SAR and modeling studies, we identified a region in these antagonists that could be modified without significant loss of activity and developed liposome targeting agents. To maximize the interactions of THI0567-targeted liposomes to α4β1 receptors on the cell surface (and based on our experience with optimizing ligand-targeted liposomes), we presented the ligand via a longer PEG chain (3400)^68^. THI0567-targeted liposomal nanoparticles bound α4β1-expressing cells with picomolar activity and a high degree of specificity and were active across multiple species (human, dog, rabbit, rat, and mouse) (Supplementary Table S3). Cross-species binding of THI0567-targeted liposomes will aid in clinically translating this approach in terms of efficacy and toxicity, and contrasts with the use of protein-targeting agents, which are often species specific.

Specific binding of THI0567-targeted liposomes to peripheral blood cell subsets, including CD11b^+^ monocytes, CD3^+^ T cells, CD19^+^ B cells, and Ly-6G^+^ neutrophils, was seen after systemic administration in healthy C57BL/6 mice. These cell types express relatively high levels of integrin α4β1, except for neutrophils, which express low levels of this integrin along with the related integrin α9β1^69^. MR imaging of THI0567-targeted liposomal-Gd in ApoE^−/−^ mice fed a high-fat diet showed enhanced signal when compared to non-targeted liposomal-Gd. In our mouse studies, the NER of THI0567-targeted liposomal-Gd was 5-fold higher than that of non-targeted liposomal-Gd in the aortic arch.

Immunofluorescent analysis of tissue sections from THI0567-targeted liposomal-Gd-treated ApoE^−/−^ mice indicated liposome localization in plaques. THI0567-targeted liposomes colocalized with the monocyte/macrophage markers CD11b and F4/80 in subendothelial regions of plaque accumulation. Notably, no THI0567-targeted liposomes were observed in endothelial or muscle cells, even though these tissues can express low levels of α4β1 and α9β1^70–72^. This specific targeting is an advantage over current PET-based approaches with ^18^F-FDG, which lack the ability to differentiate between signals generated from inflammatory cells and other cells under high metabolic demand^73^.

In the Vascular Inflammation Imaging Using Somatostatin Receptor Positron Emission Tomography (VISION) trial^24^, the ^68^Ga-labeled PET probe (^68^Ga-DOTATATE), which targets the G-protein-coupled somatostatin receptor subtype-2 on inflammatory macrophages, demonstrated superiority over ^18^F-fluorodexoyglucose (FDG) in discriminating high-risk versus low-risk atherosclerotic lesions in coronary arteries; however, the study was not powered to predict clinical events. In the coronary arteries, ^18^F-FDG imaging is inherently difficult due to complications from myocardial spill-over. Recently described PET imaging agents, such as ^18^F-Macroflor and ^68^Ga-DOTATATE, appear highly selective for tissue macrophages and macrophages with an inflammatory M1 phenotype, respectively^23,24^. However, unlike THI0567-targeted liposomes, ^18^F-Macroflor and ^68^Ga-DOTATATE do not label lymphocytes or neutrophils^23,24^. By providing the ability to image lymphocytes and neutrophils, THI0567-targeting liposomes allow for a complete quantification of overall inflammatory cell burden within plaques and a more robust imaging platform to monitor the anti-inflammatory effects of atherosclerosis drugs. The ability to image neutrophils may also be important because of the increasing evidence of neutrophil involvement in the erosion of atherosclerotic plaques^18^. THI0567-targeted liposomes cannot discriminate between different inflammatory cell subsets that express the integrin α4β1. As such, they label similar leukocyte cell subsets as does ^18^F-FDG. But, unlike ^18^F-FDG, THI0567-targeted liposomal-Gd nanoparticles are not limited by non-inflammatory cell uptake at a target site in the myocardium, do not require high doses of radiation exposure (and thus enable longitudinal studies), and are not geographically limited to high-density populations where PET tracers are available. In fact, our work demonstrates that THI0567-targeted liposomal-Gd nanoparticles can be imaged on clinical MR scanners (1T) that are readily available.

Although MR imaging has several advantages over PET/SPECT techniques, including higher spatial resolution, ease of access, and absence of radiation exposure, MR contrast sensitivity with conventional Gd compounds is relatively poor (~mM) compared with radioactive agents (nM–pM)^74^. Nanoparticle-based targeted agents were developed to improve MR contrast sensitivity. First, a liposomal-Gd platform was used to develop a highly sensitive targeted T1 contrast agent^51^, followed by the addition of THI0567 targeting. The THI0567-targeted liposomal nanoparticles contained between 3.6 × 10^4^–2.1 × 10^5^ Gd molecules per nanoparticle (varying with nanoparticle size). Additionally, presenting Gd on the surface of liposomes instead of packaging them within the core interior makes them highly effective T1 agents^51^. The liposomal-Gd agents also exhibit superior T1 relaxivity at clinically relevant MR field strengths. The classic Solomon-Blombergen-Morgan equations demonstrate an inverse relationship between MR field strength and T1 relaxivity at long rotational correlation times, which thereby improves prospects for clinical translation^75^. Although high-field strength MRI is generally preferable for molecular imaging studies in rodent models, we conducted imaging studies on a 1T MR field strength scanner to improve visualization of small features and sparse targets at clinically relevant MR field strengths, and to evaluate the feasibility for clinical translation. Here, we have shown that molecular imaging of sparse targets is indeed feasible with the use of targeted, signal-amplifying liposomal-Gd contrast agents. This work builds on our recently published work demonstrating the effectiveness of these constructs for use as molecular imaging agents at clinically relevant MR field strength^76^.

Targeting of liposomal nanoparticles to the integrin α4β1 results in their internalization. Reconstituting confocal Z-stacks into 3-dimensional images of THI0567-targeted liposomes incorporated into Jurkat cells clearly showed the internalization of the nanoparticles. In this manner, therapeutic payloads may be selectively delivered to the same α4β1-expressing cells that are being imaged. We are developing probes that could increase the therapeutic index of immunosuppressive drugs or target chemotherapeutic agents to cancerous cells in leukemia and lymphoma to combat minimal residual disease.

We have demonstrated the efficacy of THI0567-targeted liposomal-Gd as an imaging agent in the ApoE^−/−^ atherosclerotic mouse model. These findings need to be confirmed in larger animal models with more clinically relevant disease. Because THI0567-targeted liposomal-Gd binds with high affinity to multiple species, these confirmation studies should be straightforward. It is unknown whether the increased MR signals generated by THI0567-targeted liposomes are due to the recruitment of labeled inflammatory cells and/or an enhanced permeability and retention effect within the plaques. This is currently being tested.

The clearance route and biodistribution of PEGylated liposomes, either with or without targeting ligands, is well known. This has been documented since the invention of PEGylated liposomes, in numerous publications including several of our own^44,77,78^. The particles are cleared via the reticulo-endothelial system, with initial distribution to the spleen and liver, followed by sequestration of the majority of the particles in the liver in the 24-hour time frame, followed by excretion predominantly in the bile. Of particular note, there is practically zero localization in the kidneys or excretion in the urine, thus sparing the kidneys and urinary tract from any toxicity. Pathological lesions with leaky vasculature and sites of inflammation typically accumulate a small percentage of the delivered dose, between 1 and 5%. We anticipate the clearance and biodistribution of the particles in this study are consistent with those of previously studied PEGylated liposomes.

For clinical translation, the efficacy of THI0567-targeted liposome-Gd constructs will have to be demonstrated in a large animal model, such as the Watanabe heritable hyperlipidemia rabbit model of atherosclerosis. THI0567-targeted liposomes would then undergo standard investigational new drug-enabling studies, including scale-up and good manufacturing practice production, pharmacokinetics and biodistribution testing, and preclinical safety and toxicology studies. Fortunately, the preclinical development pathway for liposomes is well defined and will guide the translation to clinical development.

In summary, we have generated a high-affinity targeting ligand that specifically binds the key receptor involved in recruiting leukocytes to sites of inflammation, and particularly to inflamed atherosclerotic plaques^9–11^. When this ligand is incorporated into liposomal nanoparticles that contain a high concentration of Gd, inflammatory plaques in ApoE^−/−^ mice fed a high-fat diet can be imaged by MR on a 1 T magnet. Given the safety profile of liposomes and the ubiquity of MR imagers of this field strength, clinical translation of this agent and its adoption into use should be relatively straightforward once clinical efficacy and safety studies have been completed.

## Materials and Methods

Compound synthesis, cell adhesion assays, modelling of integrin α4β1, docking and molecular dynamics simulations, liposome formulation, and *in vitro* liposome binding assays are described in the supplement.

### Liposome binding assays

Cells were incubated with indicated concentrations of liposome in binding buffer (50 mM HEPES, pH 7.4, 150 mM NaCl, 2.5 mM KCl, 10 mM NaHCO_3,_ 1 mg/ml glucose) for 1 h at room temperature. Background nonspecific binding was determined in the presence of EDTA (20 mM). After incubation, cells were washed once in binding buffer and resuspended. Rhodamine B fluorescence was measured on a flow cytometer (LSRII, BD). Binding data are expressed as the geometric mean fluorescence intensity (gMFI). Binding Kds were generated in Prizm Software using the saturation binding equation for “One site - Total and nonspecific binding.” Total binding was fit with the equation Y = (Bmax*X/(Kd + X)) + NS*X + BKG; nonspecific binding was fit to the linear equation Y = NS*X + BKG, where X is the particle concentration of liposome, Y is Rhodamine B fluorescence, NS is nonspecific binding, and BKG is background (NS and BKG are shared). In some experiments, after *in vitro* binding assays were performed, cells were labelled with the indicated monoclonal antibodies (anti-CD64 mAb or anti-CD3 mAb OKT3) for confocal analysis. Cells were incubated with 10 ug/ml of primary antibody in FACS buffer (PBS, 10% FCS, pH 7.4) for 1 h at 4 °C. After washing, secondary GAM-FITC (2 ug/ml) was incubated with cells (1 h, 4 °C), which were then washed, subjected to cytospin onto glass coverslips, air dried, and mounted for confocal imaging.

### *In vivo* binding assays

The protocol for *in vivo* binding was approved by the Texas Heart Institute’s Institutional Animal Care and Use Committee (IACUC). All methods were performed in accordance with the relevant guidelines and regulations. C57BL/6 mice were injected with THI0567-targeted liposome, non-targeted liposome (50 pmoles Rhodamine B), or saline control via the femoral vein. Two hours after injection, we collected peripheral blood by heart puncture for cell staining and plasma for measuring liposome concentration by rhodamine B fluorescence. For staining cell subsets, heparinized peripheral blood (50 ul) was added to 50 ul PBS with 2% FBS containing Fc-Block at a final concentration of 10 ug/ml. Indicated mAbs (10 ug/ml) were added and incubated for 1 h at 4 °C. Anti-CD11b and -Ly-6G were directly conjugated with FITC. For CD3 and CD19 staining, avidin-APC-Cy7 (10 ug/ml) was added and incubated for 1 h at 4 °C. Cells were then resuspended in 2 ml of RBC lysis buffer (150 mM NH_4_Cl, 10 mM NaHCO_3_, pH 7.2) and incubated at room temperature for 7 minutes. After RBC lysis, cells were washed 2x in PBS/2%FCS. Then, the cells were run on a FACSAria cell sorter to quantify Rhodamine B fluorescence in specific cell subsets and to sort the subsets for subsequent confocal imaging.

### Magnetic resonance imaging

The protocol for animal studies was approved by the Baylor College of Medicine IACUC. All experiments were performed in accordance with the relevant guidelines and regulations. ApoE^−/−^ mice were 12–14 weeks old at the time of imaging and had been fed a high-fat diet for approximately 10 weeks. For *in-vivo* imaging studies, mice were randomized into 2 groups: (1) the targeted contrast agent group (n = 9) was administered THI0567-targeted liposomal-Gd; (2) the non-targeted contrast agent group (n = 7) was administered the control, non-targeted liposomal-Gd. The contrast agent was intravenously administered at a dose of 0.1 mmol Gd/kg in all mice. Imaging was performed on a 1.0 T permanent MRI scanner (M2 system, Aspect Technologies, Israel). A 35-mm volume coil was used to transmit and receive the radiofrequency signal. Mice were sedated with 2-3% isoflurane, placed on the MRI animal bed, and maintained under anesthesia with 1-1.5% isoflurane delivered using a nose cone. Body temperature was maintained by circulating warm water through the animal bed. Respiration rate was monitored using a pneumatically controlled pressure pad placed in the abdominal area underneath the mouse. MRI was performed at 3 time points: precontrast, immediate postcontrast (within 1 hr of contrast agent administration), and delayed postcontrast (at 72 hrs after contrast agent administration). Images were acquired using a T1-weighted 3D GRE sequence with the following scan parameters: echo time (TE) = 3.5 ms, repetition time (TR) = 20 ms, flip angle = 70, slice thickness = 0.3 mm, field of view = 54 mm, number of slices = 68, matrix = 180 × 180, NEX = 1, acquisition plane = coronal, and scan time ~6 minutes. Four consecutive scans were acquired at each time point and averaged to improve image quality.

### MR image analysis

Quantitative analysis of images was performed in Osirix (version 5.8.5, 64-bit, Pixmeo, Bernex, Switzerland) similarly as described previously^56^. Precontrast and delayed post-contrast images in both groups (non-targeted and targeted) were analyzed for signal enhancement in target regions. The signal-to-noise ratio (SNR) was determined in the region of interest (ROI) as SNR_*ROI*_ = SI_*ROI*_/SI_*noise*_, where SI_*ROI*_ is the signal intensity in the aortic vessel wall or in adjacent lumen and SI_*noise*_ is the standard deviation for a ROI drawn outside the animal. The NER was determined as in equation (1):

1 $${\rm{NER}}=[\frac{(\frac{SN{R}_{wall}}{SN{R}_{lumen}})post-(\frac{SN{R}_{wall}}{SN{R}_{lumen}})pre}{(\frac{SN{R}_{wall}}{SN{R}_{lumen}})pre}]x100 \% $$

The above analysis was performed by dividing the aorta into 3 segments: ascending aorta, aortic arch, and descending aorta (Fig. 6). Additionally, NER were determined on a per animal basis by the summation of all ROIs within each animal. For cases within the non-targeted group where signal enhancement was not clearly visible, ROIs were drawn in similar regions as those showing signal enhancement in the targeted group.

### Histology and immunofluorescence

After MR imaging, mice were euthanized, and the left ventricle was perfused via syringe with PBS/heparin (4 °C) for 10 min. Then, we removed the heart and aorta, which were fixed in 10% formalin for 30 min at room temperature. We transferred the tissues to cold PBS and dissected the heart and adipose tissue from the aorta. Then, the aorta was segmented into the root and the indicated cross-sections. The tissue segments were embedded in OCT and frozen at −80 °C. We cut 8-µm thick sections from the frozen blocks on a cryotome and mounted them on glass slides to air dry for 2 h. The sections were fixed in acetone for 10 min at −20 °C, air-dried for 15 min, and then rehydrated and permeabilized by 3 washes in PBS containing 0.05% (v/v) Tween-20 (PBST). For immunofluorescence, sections were blocked in PBS/10% goat serum (1 h, RT). Anti-CD11b-FITC, -F4/80-FITC, or -CD31, or isotype control antibodies (each at 1:100 dilution) were combined with Hoechst 33342 (1:1000), and a 100-ul solution was incubated with the tissue section overnight in a humidified chamber at 4 °C. Slides were then washed 3x in PBST. For anti-CD31 staining, donkey anti-rat IgG-FITC was added in PBST/10% goat serum for 1 h at room temperature (except for Fig. 7C, which utilized directly conjugated anti-CD31-APC-R700). After washing, the slides were sealed in mounting media with a cover slip. All images were obtained by confocal microscopy. Oil red staining was used for standard histologic evaluation of the sections.

### Statistical Analysis

All replicate data are presented as mean ± standard error of the mean (SEM) unless described otherwise. For statistical analysis of NER values from MR imaging analysis, a Kruskal-Wallis test was used. A p-value < 0.05 was considered statistically significant.

### Data and materials availability

No archived data are associated with this manuscript. THI0520, THI0550, THI0553, THI0565, THI0566, THI0567, THI0567-targeted liposomal-Gd, or non-targeted liposomal-Gd constructs, must be obtained through a material transfer agreement.

## Electronic supplementary material

Supplementary Information

Supplemental Video 1
