# Supplementary material for: Magnetic Resonance Imaging of Atherosclerotic Plaque at Clinically Relevant Field Strengths (1T) by Targeting the Integrin α4β1
Source: Sci Rep. 2018 Feb 27;8:3733. doi: 10.1038/s41598-018-21893-x (PMC5829217; doi:10.1038/s41598-018-21893-x)

Magnetic resonance imaging of atherosclerotic plaque at clinically relevant field strengths (1T) by targeting the integrin α4β1

Darren G. Woodside[1]*, Eric A. Tanifum[2], Ketan B. Ghaghada[2], Ronald J. Biediger[1], Amy R. Caivano[1], Zbigniew A. Starosolski[2], Sayadeth Khounlo[1], Saakshi Bhayana[2], Shahrzad Abbasi[1], John W. Craft Jr.[1,3], David S. Maxwell[4†], Chandreshkumar Patel[2], Igor V. Stupin[2], Deenadayalan Bakthavatsalam[1], Robert V. Market[1], James T. Willerson[5], Richard A.F. Dixon[1], Peter Vanderslice[1], and Ananth V. Annapragada[2]*

[1]Department of Molecular Cardiology, Texas Heart Institute, 6770 Bertner Avenue, Houston, Texas 77030, USA. [2]Department of Pediatric Radiology, Texas Children's Hospital, 6621 Fannin Street, Houston, Texas 77030, USA. [3]Department of Biology and Chemistry, University of Houston, 4800 Calhoun Road, Houston, Texas, 77004, USA. [4]Department of Experimental Therapeutics, University of Texas MD Anderson Center, 1515 Holcombe Boulevard, Houston, Texas 77030, USA. [5]Division of Cardiology Research, Texas Heart Institute, 6770 Bertner Avenue, Houston, Texas 77030, USA.

Supplementary Materials

Supplementary Materials and Methods

Supplementary Table S1. Selectivity data of antagonists tested against a panel of integrin targets.

Supplementary Table S2. Constituents of THI0567-targeted and non-targeted liposomal-Gd constructs.

Supplementary Table S3. Binding affinities of THI0567-targeted liposomal-Gd against rat, rabbit, and dog cells.

Supplementary Figure S1. Examples of cell-based binding assays.

Supplementary Figure S2. Expression of integrin α4β1 on different cell lines and mutants.

Supplementary Figure S3. Binding of targeted liposomes in the presence of serum.

Supplementary Figure S4. Synthetic scheme 1 describing synthesis of compounds 1 through 12.

Supplementary Figure S5. Synthetic scheme 2 describing synthesis of compounds 13 through 19.

Supplementary Figure S6. Synthetic scheme 3 describing synthesis of compounds 20 through 25.

Supplementary Figure S7. Synthetic scheme 4 describing synthesis of compounds 24 through 30.

Supplementary Figure S8. Structure of compound 31.

Supplementary Figure S9. THI0565 geometry for docking.

Supplementary Figure S10. THI0565 unbiased docking.

Supplementary Figure S11. Molecular dynamics simulations comparing docked and endpoint frame.

Supplementary Movie S1. THI0567 uptake by Jurkat T cells.

Materials and Methods

Compound synthesis

Synthesis of Compounds THI0520 (**25**), THI0553 (**31**), THI0550 (**30**), THI0565 (**12**), THI0566 (**17**), and THI0567 (**19**) are described in separate headings below.

The synthetic scheme to generate Compounds **1** through **12** is shown in Figure S4**.** For compound **1**: To a solution of ethylene glycol mono *tert*-butyl ether (3.44 mL, 3.096 g, 26.2 mmol) in anhydrous tetrahydrofuran (26.2 mL) at 0°C under argon, sodium hydride (60% dispersion in mineral oil, 655 mg, 16.4 mmol) was added in several portions. The mixture was stirred at 0°C for 30 minutes, and 2-chloro-6-fluorobenzonitrile (2.028 g, 13.1 mmol) was added. The reaction mixture was allowed to slowly warm to room temperature and was stirred overnight. The mixture was diluted with a 1:1 mixture of hexanes:ethyl acetate and washed with water (twice) and brine. The organic layer was dried over magnesium sulfate, filtered, and concentrated under reduced pressure. The residue was purified by automated silica gel chromatography (Biotage®, SNAP 50 KP-Sil, 0-50% ethyl acetate in hexanes) to give **1** (3.22 g) as a colorless oil. ^{1}H NMR (300 MHz, $CDCl_3$): δ 7.43 (dd, J = 8.4, 8.3 Hz, 1H), 7.07 (dd, J = 8.3, 0.8 Hz, 1H), 6.94 (dd, J = 8.4, 0.8 Hz, 1H), 4.21 (t, J = 5.3 Hz, 2H), 3.79 (t, J = 5.3 hZ, 2H), 1.25 (s, 9H).

Compound **2**: To a solution of **1** (3.21 g, 12.7 mmol) in anhydrous tetrahydrofuran (94 mL) at 0 °C under argon, lithium aluminum hydride (2.0 M in tetrahydrofuran, 12.7 mL, 25.4 mmol) was added dropwise by syringe. The mixture was allowed to gradually warm to room temperature and was stirred overnight. The mixture was re-cooled to 0°C, then water (0.97 mL) was added dropwise by syringe. The mixture was vigorously stirred for 10 minutes. Then a solution of sodium hydroxide (20% by weight in water, 0.71 mL) was added dropwise, and

stirring was continued for 10 minutes. Finally, water (3.53 mL) and Celite® filter aid were added. The mixture was diluted with diethyl ether, vigorously stirred for 30 minutes, and then filtered through additional Celite®, washing with ether. The filtrate was concentrated under reduced pressure to give **2** (3.27 g) as a pale yellow oil. This material was used without purification. ^{1}H NMR (300 MHz, CD_3SOCD_3): δ 7.19 (t, J = 8.3 Hz, 1 H), 6.98 (dd, J = 8.3, 0.9 Hz, 1H), 6.96 (dd, J = 8.3, 0.9 Hz, 1H), 4.08 (m, 2H), 3.77 (s, 2H), 3.65 (m, 2H), 1.70 (br. s, 2H), 1.16 (s, 9H).

Compound **4**: To a flask containing **3** (prepared according to the procedures described in Step One of Example 25 in US 6972296[1], 2.11g, 13.3 mmol), a solution of **2** (3.26 g, 12.7 mmol) in methanol (50 mL) was added along with a methanol rinse (14 mL). The resulting mixture was heated to 45°C overnight and then refluxed for 24 hours. The mixture was cooled to room temperature and concentrated under reduced pressure. The residue was taken up in ethyl acetate and concentrated (twice). The residue was then suspended in ethyl acetate (30 mL), stirred for 30 minutes, and then filtered, washing with ethyl acetate. The solid was dried under vacuum to give **4** (1.47 g) as a light yellow powder. ^{1}H NMR (300 MHz, CD_3SOCD_3): δ 10.54 (br. s, 1H), 7.35 (t, J = 8.3 Hz, 1 H), 7.09 (d, J = 8.3 Hz, 1H), 7.09 (d, J = 8.3 Hz, 1H), 6.79 (q, 0.8 Hz, 1H), 5.63 (s, 1H), 5.01 (s, 2H), 4.09 (t, J = 4.9 Hz, 2H), 3.58 (t, J = 4.9 Hz, 2H), 1.76 (d, J = 0.8 Hz, 3H), 1.10 (s, 9H).

Compound **5**[2]: To a suspension of **4** (1.789 g, 4.89 mmol) in tetrahydrofuran (25 mL) at room temperature open to air, *tert*-butyl nitrite (1.74 mL, 14.7 mmol) was added. The resulting mixture was stirred overnight, then diluted with methanol (10 mL), and concentrated under reduced pressure to give **5** as a dark yellow oil. This material was used without purification. ^{1}H NMR (300 MHz, CD_3SOCD_3): δ 12.11 (br. s, 1H), 7.36 (t, J = 8.3 Hz, 1 H), 7.25 (q, J = 0.9 Hz,

1H), 7.10 (d, J = 8.3 Hz, 1H), 7.09 (d, J = 8.3 Hz, 1H), 5.10 (s, 2H), 4.07 (t, J = 5.1 Hz, 2H), 3.57 (t, J = 5.1 Hz, 2H), 1.92 (d, J = 0.9 Hz, 3H), 1.10 (s, 9H).

Compound **6**: To a solution of crude **5** (4.89 mmol theoretical from step four) in N,N-dimethylformamide (DMF, 16.3 mL), zinc dust (1.44 g, 22.0 mmol) and triethylamine hydrochloride (3.702 g, 26.9 mmol) were added. The mixture was heated to 55°C for 3 hours, cooled to room temperature, and 1,1'-carbonyldiimidazole (2.379 g, 14.7 mmol) was added in a single portion. The mixture was heated to 80°C for 1.5 hours and then was cooled to room temperature. The crude reaction mixture was filtered into a flask containing water (200 mL) washing with a small amount of DMF. The resulting suspension was filtered, washing the collected solid with water. The solid was then partitioned between dichloromethane and aqueous HCl (2N). The aqueous layer was extracted twice with dichloromethane; the combined organic layers were washed with water, then dried over magnesium sulfate, filtered and concentrated to give **6** (1.91 g) as an orange-tan solid. This material was used without purification. ^{1}H NMR (300 MHz, CD_3SOCD_3): δ 12.02 (br. s, 1H), 7.38 (t, J = 8.3 Hz, 1 H), 7.11 (d, J = 8.3 Hz, 2H), 6.89 (q, J = 0.9 Hz, 1H), 5.21 (s, 2H), 4.09 (t, J = 5.0 Hz, 2H), 3.56 (t, J = 5.0 Hz, 2H), 2.00 (d, J = 0.9 Hz, 3H), 1.07 (s, 9H).

Compound **7**[3]: To a solution of N,N-dimethyaminopyridine (DMAP) (200 mg, 1.64 mmol) in DMF (41 mL) at room temperature under argon, *tert*-butyl hydrogen malonate (3.94 g, 24.6 mmol) and 3-hydroxybenzaldehyde (2.00 g, 16.4 mmol) were added. Piperazine (0.16 mL, 1.64 mmol) was added, and the mixture was stirred at room temperature for 3 days. Thin layer chromatography (TLC) indicated significant starting material remained, so the mixture was heated to 55°C overnight and then cooled to room temperature. The mixture was diluted with water (200 mL) and was extracted with 4:1 hexanes:ethyl acetate. The organic layer was washed

with saturated aqueous ammonium chloride, water (twice), and brine, and then was dried over magnesium sulfate, filtered, and concentrated under reduced pressure to give **7** (3.73 g) as a white solid. This material contained trace residual solvent but was used without further purification. ^{1}H NMR (300 MHz, CD_3SOCD_3): δ 9.60 (s, 1H), 7.45 (d, J = 16.2 Hz, 1H) 7.20 (t, J = 8.0 Hz, 1 H), 7.10 (d, J = 8.0 Hz, 1H), 6.99 (t, J = 1.1 Hz, 1H), 6.81 (m, 1H), 6.38 (d, 16.2 Hz, 1H), 1.48 (s, 9H).

Compound **8**: To a solution of **7** (2.04 g, 9.26 mmol) in dichloromethane (46 mL) at 0°C under argon, ethylene glycol mono *tert*-butyl ether (1.58 mL, 12.04 mmol) was added by syringe followed by triphenylphosphine (3.65 g, 13.9 mmol). The mixture was stirred 10 minutes, and then a solution of diethyl azodicarboxylate (40% by weight in toluene, 6.33 mL, 13.9 mmol) was added. After the mixture was stirred at room temperature overnight, TLC indicated partial conversion. Additional portions of ethylene glycol mono *tert*-butyl ether (0.79 mL, 6.0 mmol), triphenylphosphine (1.82 g, 7.0 mmol), and diethyl azodicarboxylate (3.2 mL, 7.0 mmol) were added. The mixture was stirred an additional 24 hours and then was concentrated under reduced pressure. The residue was taken up in 3:1 hexanes:ethyl acetate, and the solution was washed with water (twice) and brine. The organic layer was dried over magnesium sulfate, filtered and concentrated under reduced pressure. The residue was taken up in 9:1 hexanes:dichloromethane with heating to give a clear solution that was allowed to cool to room temperature and stand overnight. Some crystals adhered to the side of the flask, which were by-products from the reaction. The solution was decanted and applied directly to a 5-inch plug of silica gel, which was sequentially eluted with 9:1 hexanes:dichloromethane, 19:1 hexanes:ethyl acetate, 9:1 hexanes:ethyl acetate, and finally 3:1 hexanes:ethyl acetate to give **8** (2.029 g) as a pale yellow oil. A small amount of unreacted starting material (0.39 g) was also isolated. ^{1}H NMR (300

MHz, CD_3SOCD_3): δ 7.51 (d, J = 16.2 Hz, 1H), 7.20-7.33 (m, 3H), 6.97 (m, 1H), 6.56 (d, J = 16.2 Hz, 1H), 4.07 (m, 2H), 3.63 (m, 2H), 1.48 (s, 9H), 1.16 (s, 9H).

Compound **9**: To a solution of (R)-(+)-N-benzyl-α-methylbenzylamine (1.77 g, 8.40 mmol) in tetrahydrofuran (28 mL) cooled to -78°C under argon, n-butyllithium (1.6 M in hexanes, 4.88 mL, 7.80 mmol) was added dropwise over 10 minutes. The bright red solution was stirred at

-78°C for 30 minutes, and a solution of **8** (2.029 g, 6.33 mmol) in tetrahydrofuran (8 mL) was added dropwise by syringe along with a THF (2 mL) rinse. The resulting solution was stirred at

-78°C for 4 hours, and absolute ethanol (3 mL) was added, followed by saturated aqueous ammonium chloride (25 mL). The resulting mixture was allowed to warm until the ice had completely melted and then was extracted with 3:1 hexanes:ethyl. The organic layer was washed with water (twice) and a 1:1 mixture of saturated aqueous sodium bicarbonate:water and brine, and then dried over magnesium sulfate (anhydrous), filtered, and concentrated under reduced pressure. The residue was purified by automated chromatography on silica gel (Biotage®, SNAP100 KP-Sil, eluting with 5-20% ethyl acetate in hexanes) to give **9** (2.98 g) as a colorless viscous oil. ^1H NMR (300 MHz, CD_3SOCD_3): δ 7.10-7.47 (m, 11H), 6.95 (d, J = 7.8 Hz, 1H), 6.91 (s, 1H), 6.81 (dd, J = 8.1, 2.1 Hz, 1H), 4.15 (dd, J = 9.6, 5.1 Hz, 1H), 3.93-4.07 (m, 3H), 3.54-3.7 (m, 4H), 2.53-2.68 (m, 2H), 1.19 (s, 9H), 1.16 (s, 9H), 1.09 (d, J = 6.9 Hz, 3H).

Compound **10**: To a solution of **9** (2.97 g, 5.80 mmol) in absolute ethanol (39 mL) at room temperature under argon, glacial acetic acid (0.5 mL), palladium metal on carbon (Degussa type E101 NE/W, 50% H_2O, 10% Pd dry weight basis, 0.98 g, 0.46 mmol Pd). The atmosphere was replaced with hydrogen (toggling between vacuum and hydrogen from a balloon several

times), and the reaction was stirred overnight. The mixture was filtered through Celite®, washing with ethanol, and the filtrate was concentrated under reduced pressure. The residue was recrystallized from diethyl ether and hexanes to give **10** (1.373 g) as a white crystalline solid. No further attempts were made to isolate additional material from the mother liquor. ^{1}H NMR (300 MHz, CD_3SOCD_3): δ 7.18 (t, J = 8.0 Hz, 1H), 6.94 (d, J = 1.8 Hz, 1H), 6.89 (D, J = 8.0 Hz, 1H), 6.76 (dd, J = 8.0, 1.8 Hz, 1H), 5.34 (very br. s, 3 H), 4.10 (t, J = 7.1 Hz, 1H), 4.00 (t, J = 5.0 Hz, 2H), 3.62 (t, J = 5.0 Hz, 2H), 2.48 (m, 2H), 1.90 (s, 3H), 1.32 (s, 9H), 1.16 (s, 9H).

Compound **11**: A solution of **6** (1.003 g, 2.46 mmol) and **10** (890 mg, 2.24 mmol) in DMF (12.3 mL) and N,N-diisopropylethylamine (DIPEA) (0.59 mL, 3.36 mmol) under argon was heated to 55°C for 8 hours. An aliquot indicated unreacted **10**, so additional **6** (100 mg, 0.25 mmol) was added. Then, the mixture was heated to 55°C overnight, cooled to room temperature, and diluted with 1:1 hexanes:ethyl acetate and HCl (2N). The organic layer was washed with water (3 times) and brine, dried over $MgSO_4$ and filtered. The filtrate was concentrated under reduced pressure, and the resulting residue was purified automated chromatography on silica gel (Biotage®, SNAP100 KP-Sil, eluting with 25-50% ethyl acetate in hexanes). A few fractions containing the desired product also contained an impurity. The fractions were concentrated and repurified (Biotage®, SNAP10 Ultra, eluting with 30-50% ethyl acetate in hexanes). Fractions from both separations containing only the desired product were combined and concentrated to give **11** (1.25 g) as a pale yellow foam. This material contained approximately 4% ethyl acetate by weight but was used as is. ^{1}H NMR (300 MHz, CD_3SOCD_3): δ 12.82 (s, 1H), 8.31 (br. s overlapping d, 2H), 7.34 (t, J = 8.3 Hz, 1H), 7.24 (t, J = 8.1 Hz, 1H), 7.04-7.12 (m, 2H), 6.80-6.91 (m, 3H), 6.73 (q, J = 0.9 Hz, 1H), 5.15 (d, J = 13.8 Hz, 1H), 5.09 (d, J = 13.8 Hz, 1H), 5.03 (m, 1H), 3.87-4.13 (m, 4 H), 3.62 (t, J = 4.8 Hz, 2H), 3.55 (t, J = 5.1 Hz, 2H), 2.70 (dd, J = 15.0,

6.3 Hz, 1H), 2.61 (dd, J = 15.0, 8.6 Hz, 1H), 1.82 (d, J = 0.9 Hz, 3H), 1.32 (s, 9H), 1.15 (s, 9H), 1.07 (s, 9H).

Compound **12**: To a solution of **11** (26 mg, 0.035 mmol) in dichloromethane (0.2 mL) at room temperature, trifluoroacetic acid (0.2 mL) was added. The mixture was stirred at room temperature for 4 hours and then was concentrated. The residue was dissolved in dichloromethane and concentrated. The residue was then taken up in a 1:1 mixture of acetonitrile and water (2 mL) and allowed to stand overnight. The resulting mixture was diluted with water (2 mL) and then was frozen in a dry ice/acetone bath and lyophilized to give **12** (19.6 mg) as a white powder. ^{1}H NMR (300 MHz, CD_3SOCD_3): δ 8.31 (br. s overlapping d, 2H), 7.32 (t, J = 8.1 Hz, 1H), 7.23 (t, J = 8.1 Hz, 1H), 6.98-7.11 (m, 2H), 6.94 (q, J = 0.9 Hz, 1H), 6.78-6.89 (m, 3H), 5.17 (s, 2H), 5.02 (m, 1H), 4.86 (br. s, 2H), 4.01 (t, J = 4.8 Hz, 2H), 3.96 (t, J = 5.1 Hz, 2H), 3.62-3.76 (m, 4H), 2.55-2.71 (m 2H), 1.84 (d, J = 0.9 Hz, 3H).

The synthetic scheme to generate Compounds **13** through **19** is shown in Figure S5. Compound **13**[4]: To a solution of bis-1,2-(2-iodoethoxy)ethane (7.37 g, 19.9 mmol) in DMF (133 mL) at room temperature under argon, sodium azide (1.29 g, 19.9 mmol) and tetraethylammonium iodide (257 mg, 1.00 mmol) were added. The resulting mixture was stirred at room temperature overnight, diluted with water (400 mL), and extracted with 9:1 hexanes:ethyl acetate (100 mL 3 times). The organic layers were combined, washed with water and brine, dried over magnesium sulfate, filtered, and concentrated under reduced pressure. This gave approximately a statistical mixture of starting material, the desired product, and the bis-azide. This mixture was purified by automated chromatography on silica gel (Biotage®, SNAP100 KP-Sil, eluting with 10-15% ethyl acetate in hexanes) to give **13** (1.95 g) as a yellow

oil. NMR (300 MHz, CD_3SOCD_3): δ 3.67 (t, J = 6.3 Hz, 2H), 3.61 (t, J = 4.8 Hz, 2H), 3.57 (s, 4H), 3.93 (t, J = 4.8 Hz, 2H), 3.21 (t, J = 6.3 Hz, 2H).

Compound **14**: To a solution of **11** (1.25 g, 96% by weight, 1.61 mmol) and **13** (918 mg, 3.22 mmol) in DMF (8 mL) at room temperature under argon, potassium carbonate (668 mg, 4.83 mmol) was added. The resulting mixture was heated to 80°C overnight, cooled to room temperature, diluted with ethyl acetate, and washed with water (3 times) and brine. The organic layer was dried over magnesium sulfate, filtered, and concentrated under reduced pressure. The residue was purified by automated chromatography on silica gel (Biotage®, SNAP100 KP-Sil, eluting with 50-100% ethyl acetate in hexanes) to give **14** (1.16 g) as a light yellow viscous oil. NMR (300 MHz, CD_3SOCD_3): δ 7.36 (t, J = 8.3 Hz, 1H), 7.32 (s, 1H), 7.22 (t, J = 8.0 Hz, 1H), 7.09 (m, 2H), 6.98 (d, J = 8.7 Hz, 1H), 6.86-6.92 (m, 2H), 6.77-6.83 (m, 2H), 5.10 (d, J = 14.1 Hz, 1H), 5.04 (d, J = 14.1 Hz, 1H), 5.01 (m, 1H), 3.97-4.16 (m, 6H), 3.44-3.67 (m, 12H), 3.30-3.37 (m, 4H), 2.58-2.74 (m, 2H), 1.83 (d, J = 0.6 Hz, 3H), 1.30 (s, 9H), 1.16 (s, 9H), 1.10 (s, 9H).

Compound **15**: To a solution of **14** (1.16 g, 1.29 mmol) in THF (12.9 mL) at room temperature under argon, triphenylphosphine (508 mg, 1.94 mmol) was added. The mixture was stirred for 1.5 hours, water (0.26 mL) was added, and stirring was continued overnight. The mixture was concentrated, and the residue was purified by automated chromatography on silica gel (Biotage®, SNAP25 KP-Sil, eluting with 75-100% ethyl acetate in hexanes, then 0-10% methanol with 2% added triethylamine in ethyl acetate, then 10-20% methanol with 2% added triethylamine in dichloromethane) to give **15** (1.101 g) as a brownish yellow viscous oil. NMR (300 MHz, CD_3SOCD_3): δ 7.36 (t, J = 8.3 Hz, 1H), 7.32 (s, 1H), 7.22 (t, J = 7.8 Hz, 1H), 7.09 (m, 2H), 6.98 (d, J = 8.7 Hz, 1H), 6.84-6.93 (m, 2H), 6.75-6-84 (m, 2H), 5.10 (d, J = 14.1 Hz,

1H), 5.04 (d, J = 14.1 Hz, 1H), 4.99 (m, 1H), 3.97-4.17 (m, 6H), 3.40-3.67 (m, 10H), 3.30 (t, J = 5.7 Hz, 2H), 2.58-2.74 (m, 2H), 2.61 (t, J = 5.7 Hz, 2H), 1.83 (s, 3H), 1.30 (s, 9H), 1.16 (s, 9H), 1.10 (s, 9H).

Compound **16**: To a solution of **15** (1.10 g, 1.26 mmol) in DMF (6.3 mL) at room temperature under argon, DIPEA (0.66 mL, 3.78 mmol) and diglycolic anhydride (439 mg, 3.78 mmol) were added. The mixture was stirred at room temperature overnight, diluted with ethyl acetate, and washed with aqueous HCl (2N), water (3 times), and brine. The organic layer was dried over magnesium sulfate, filtered, and concentrated under reduced pressure. The residue was purified by automated reverse-phase chromatography (Biotage®, SNAP30 C18, eluting with 50-100% acetonitrile in water). Fractions containing **16** were combined, and the acetonitrile was removed by rotary evaporation until a cloudy solution resulted. Enough acetonitrile was added to give a clear solution, which was frozen in a dry ice/acetone bath and lyophilized to give **16** (1.00 g) as a fluffy white powder. NMR (300 MHz, CD_3SOCD_3): δ 12.81 (br. s, 1H), 7.82 (t, J = 5.3 Hz, 1H), 7.35 (t, J = 8.3 Hz, 1H), 7.33 (s, 1H), 7.21 (t, J = 7.8 Hz, 1H), 7.09 (m, 2H), 6.99 (d, J = 8.7 Hz, 1H), 6.84-6.93 (m, 2H), 6.75-6-84 (m, 2H), 5.10 (d, J = 14.1 Hz, 1H), 5.04 (d, J = 14.1 Hz, 1H), 4.99 (m, 1H), 3.97-4.17 (m, 8H), 3.94 (s, 2H), 3.63 (t, J = 4.8 Hz, 2H), 3.58 (t, J = 5.0 Hz, 2H), 3.33-3.52 (m, 8 H), 3.22 (m, 2H), 2.58-2.74 (m, 2H), 1.83 (d, J = 0.9 Hz, 3H), 1.30 (s, 9H), 1.16 (s, 9H), 1.10 (s, 9H).

Compound **17**: To a flask containing **16** (4.7 mg, 0.0047 mmol), dichloromethane (0.3 mL) and trifluoroacetic acid (0.3 mL) were added. The flask was sealed with a septum and stirred at room temperature for 4 hours and then concentrated. The residue was dissolved in dichloromethane and re-concentrated (3 times). The residue was then taken up in a 1:1 mixture of acetonitrile and water and allowed to stand overnight. The resulting mixture was frozen in a

dry ice/acetone bath and lyophilized to give **17** (4.2 mg) as a fluffy white powder. NMR (300 MHz, CD_3OD): δ 7.34 (t, J = 8.1 Hz, 1H), 7.26 (t, J = 7.8 Hz, 1H), 7.10 (dd, J = 8.1, 0.6 Hz, 1H), 6.93-7.05 (m, 4H), 6.85 (dd, J = 8.1, 2.0 Hz, 1H), 5.37 (s, 2H), 5.24 (t, J = 6.6Hz, 1H), 4.24 (m, 2H), 4.14 (s, 2H), 3.97-4.12 (m, 6H), 3.88 (t, J = 4.7 Hz, 2H), 3.77 (t, J = 4.4 Hz, 2H), 3.66 (t, J = 4.7 Hz, 2H), 3.48-3.63 (m, 6H), 3.40 (t, J = 5.4 Hz, 2H), 2.84 (m, 2H), 1.95 (s, 3H).

Compound **18**: To a solution of **16** (580 mg, 0.585 mmol) in DMF (5.9 mL) at room temperature under argon, DIPEA (0.38 mL, 2.19 mmol) and N,N,N',N'-tetramethyl-O-(N-succinimidyl)uranium tetrafluoroborate (TSTU) (283 mg, 0.73 mmol) were added. The resulting mixture was stirred for 1.5 hours and then was cannulated, along with a DMF (0.5 mL) rinse, into a solution of DSPE-PEG_{3400}-NH_2 (Laysan Bio, 616 mg, approximately 0.146 mmol) in DMF (14.6 mmol) at room temperature under argon. The resulting mixture was stirred for 2.5 days and then was concentrated under reduced pressure. The residue was taken up in toluene and concentrated several times; then it was taken up in acetonitrile and water. Acetonitrile was removed by rotary evaporation until the solution was slightly cloudy, and then acetonitrile was added dropwise until a clear solution resulted. The mixture was frozen in a dry ice/acetone bath and lyophilized. The resulting powder was purified by size-exclusion chromatography (Sephadex LH-20) in 4 portions, eluting with methanol. Individual fractions were spotted on TLC plates (with UV indicator) and visualized by UV light as well as a phosphomolybdic acid (PMA) stain (10% in ethanol). The material that was both UV and PMA active came off the column in 2 bands. For the first band, the spots on the TLC plate were very compact with minimal spreading beyond the tip of the micropipet spotter. For the second band, the spots were more diffuse, with spreading all the way to the edge of the spotting solvent front. The first band contained the

desired product, and fractions from this band were combined and concentrated under reduced pressure to give **18** (690 mg) as a tan-yellow glass.

Compound **19**: To a solution of **18** (680 mg, approximately 0.13 mmol) in dichloromethane (9 mL), trifluoroacetic acid (9 mL) was added. The mixture was stirred at room temperature for 4 hours and then was concentrated. The residue was dissolved in dichloromethane and concentrated (5 times). The residue was then taken up in a 1:1 mixture of acetonitrile and water (30 mL) and allowed to stand overnight. The resulting mixture was diluted with water (60 mL), and then the resulting mixture was frozen in a dry ice/acetone bath and lyophilized. The resulting powder was purified by size-exclusion chromatography (Sephadex LH-20) in 2 portions, eluting with methanol. Fractions were spotted as described above, and fractions containing material that was both UV and PMA active were combined and concentrated. The residue was taken up in water (50 mL) and acetonitrile (15 mL), and the resulting mixture was frozen in a dry ice/acetone bath and lyophilized to give **19** (572 mg) as an off-white solid. MALDI (Positive mode, sinapic acid): central mass of distribution: 4867.8.

The synthetic scheme to generate Compounds **20** through **25** is shown in Figure S6. Compound **20**: To a suspension of 3-ethoxycinnamic acid (2.028 g, 10.6 mmol) in toluene (13.3 mL) at room temperature under argon, *tert*-butyl 2,2,2-trichloroacetimidate (2.37 mL, 13.3 mmol) was added. The mixture was heated to 50°C overnight, at which time TLC analysis revealed partial conversion. Additional *tert*-butyl 2,2,2-trichloroacetimidate (1.2 mL) was added, and heating was continued for 24 hours. The reaction was still not complete, so more *tert*-butyl 2,2,2-trichloroacetimidate (1.2 mL) was added, and heating was continued for an additional 24 hours. The resulting mixture was filtered, washing with toluene, and the filtrate was concentrated under reduced pressure. The residue was purified by chromatography on silica gel, eluting with

10% ethyl acetate in hexanes to give **20** (2.22 g) as a colorless oil. NMR (300 MHz, CD_3SOCD_3): δ 7.51 (d, J = 16.1 Hz, 1H), 7.30 (t, J = 7.8 Hz, 1H), 7.20-7.26 (m, 2H), 6.95 (ddd, J = 7.8, 2.6, 1.0 Hz, 1H), 6.54 (d, J = 16.1 Hz, 1H), 4.06 (q, J = 7.0 Hz, 2H), 1.48 (s, 9H), 1.32 (t, J = 7.0 Hz, 3H).

Compound **21**: Following the general procedure for the Michael addition, (R)-(+)-N-benzyl-α-methylbenzylamine (1.143 g, 5.41 mmol) in tetrahydrofuran (18 mL) cooled to -78°C under argon was deprotonated with n-butyllithium (1.6 M in hexanes, 3.17 mL, 5.10 mmol). After stirring for 30 minutes at -78°C, a solution of **20** (840 mg, 3.38 mmol) in tetrahydrofuran (5.6 mL) was added. The solution was stirred an additional 4 hours, and the reaction was quenched with absolute ethanol (2 mL), followed by saturated aqueous ammonium chloride (25 mL). Purification by automated silica gel chromatography (Biotage®, SNAP100 KP-Sil, eluting with 5-20% ethyl acetate in hexanes) gave **21** (1.51 g) as a colorless viscous oil. NMR (300 MHz, CD_3SOCD_3): δ 7.39-7.46 (m, 2H), 7.31-7.39 (m, 2H), 7.21-7.29 (m, 6H), 7.17 (m, 1H), 6.94 (d, J = 7.8 Hz, 1H), 6.89 (br. s, 1H), 6.80 (dd, J = 8.0, 2.0 Hz, 1H), 4.14 (dd, J = 9.6, 5.2 Hz, 1H), 3.93-4.07 (m, 3H), 3.66 (d, J = 15.2 Hz, 1H), 3.60 (d, J = 15.2 Hz, 1H), 2.63 (dd, J = 14.9, 5.2 Hz, 1H), 2.53 (dd, J = 14.9, 9.6 Hz, 1H), 1.33 (t, J = 6.9 Hz, 3H), 1.19 (s, 9H), 1.09 (d, J = 6.9 Hz, 3H).

Compound **22**: Following the general procedure for catalytic hydrogenolysis of the benzyl groups, a mixture of **21** (1.50 g, 3.26 mmol), palladium metal on carbon (Degussa type E101 NE/W, 50% H_2O, 10% Pd dry weight basis, 0.55 g, 0.26 mmol Pd), and glacial acetic acid (0.2 mL) in absolute ethanol (22 mL) was stirred under a hydrogen atmosphere overnight. After undergoing filtering and concentrating, the residue was taken up in a 1:1 mixture of ethyl acetate and hexanes and was washed with aqueous sodium hydroxide, water, and brine. The organic

phase was dried over magnesium sulfate, filtered, and concentrated to give **22** (798 mg) as a light-yellow oil. NMR (300 MHz, CD_3SOCD_3): δ 7.17 (t, J = 7.8 Hz, 1H), 6.86-6.94 (m, 2H), 6.74 (ddd, J = 7.8, 2.7, 0.9 Hz, 1H), 4.09 (t, J = 7.1 Hz, 1H), 4.00 (q, J = 6.9 Hz, 2H), 2.37-2.53 (m, 2H), 1.93 (br. s, 2H), 1,27-1.37 (m, 12H).

Compound **24**: Following the general procedure for urea formation, a solution of **22** (146 mg, 0.55 mmol) and **23** (US 6972296, compound **151**, Example 36 [1], 166 mg, 0.50 mmol) in DMF was heated to 55°C overnight. No DIPEA was added since **22** was the freebase. Purification by automated silica gel chromatography (Biotage®, SNAP100 KP-Sil, eluting with 30-40% ethyl acetate in hexanes) gave **25** (252 mg) as an off-white foam. NMR (300 MHz, CD_3SOCD_3): δ 12.79 (s, 1H), 8.32 (s, 1H), 8.28 (d, J = 8.4 Hz, 1H), 7.31 (t, J = 8.4 Hz, 1H), 7.24 (t, J = 8.1 Hz, 1H), 7.05 (dd, J = 8.1, 0.9 Hz, 1H), 6.99 (d, J = 8.4 Hz, 1H), 6.77-6.90 (m, 4H), 5.11 (s, 2H), 5.02 (m, 1H), 3.95-4.07 (m, 4H), 2.69 (dd, J = 15.0, 6.3 Hz, 1H), 2.60 (dd, J = 15.0, 8.7 Hz, 1H), 1.84 (d, J = 0.9 Hz, 3H), 1.26-1.36 (m, 12H), 1.24 (t, J = 6.9 Hz, 3H).

Compound **25**: A solution of **24** (24 mg, 0.040 mmol) in dichloromethane (0.2 mL) and trifluoracetic acid (0.2 mL) was stirred at room temperature for 1 hour and then was concentrated. The residue was dissolved in dichloromethane and concentrated (3 times). The residue was dissolved in a 1:1 mixture of acetonitrile and water (2 mL). Water was added dropwise until the solution became slightly cloudy (approximately 3 mL). Acetonitrile was added dropwise until a clear solution resulted, which was frozen in a dry ice/acetone bath and lyophilized to give **25** (20.2 mg) as a fluffy white powder. NMR (300 MHz, CD_3SOCD_3): δ 12.78 (s, 1H), 12.36 (br. s, 1H), 8.32 (s, 1H), 8.29 (d, J = 8.1 Hz, 1H), 7.31 (t, J = 8.4 Hz, 1H), 7.23 (t, J = 8.1 Hz, 1H), 7.05 (dd, J = 8.1, 0.9 Hz, 1H), 6.99 (d, J = 8.4 Hz, 1H), 6.78-6.85 (m, 4H), 5.11 (s, 2H), 5.02 (m, 1H), 4.01 (q, J = 6.9 Hz, 2H), 4.00 (q, J = 6.9 Hz, 2H), 2.72 (dd, J =

15.6, 6.0 Hz, 1H), 2.62 (dd, J = 15.6, 8.3 Hz, 1H), 1.84 (s, 3H), 1.31 (t, J = 6.9 Hz, 3H), 1.25 (t, J = 6.9 Hz, 3H).

The synthetic scheme to generate Compounds **24** through **30** is shown in Figure S7.Compound **26**: Following the general procedure for alkylation of the pyridone hydroxyl, a mixture of **24** (710 mg, 1.18 mmol), tert-butyl (6-bromohexyl)carbamate (992 mg, 3.54 mmol) and potassium carbonate (326 mg, 2.36 mmol) in dimethylformamide (4 mL) was heated to 80°C overnight. Purification by automated silica gel chromatography (Biotage®, SNAP100 KP-Sil, eluting with 30-40% ethyl acetate in hexanes) gave **26** (790 mg) as a colorless oil. NMR (300 MHz, CD_3SOCD_3): δ 7.33 (t, J = 8.4 Hz, 1H), 7.26 (s, 1H), 7.20 (t, J = 8.1 Hz, 1H), 7.06 (dd. J = 8.1, 0.9 Hz, 1H), 7.01 (d, J = 8.4 Hz, 1H), 6.93 (d, J = 8.7 Hz, 1H) 6.83-6.89 (m, 3H), 6.73-6.81 (m, 2H), 5.08 (d, J = 14.3 Hz, 1H), 5.03 (d, J = 14.3 Hz, 1H), 4.98 (m, 1H), 3.84-4.08 (m, 6H), 2.86 (m, 2H), 2.64 (m, 2H), 1.83 (d, J = 0.6 Hz, 3H), 1.13-1.54 (m, 32H).

Compound **27**: To a solution of **26** in ethyl acetate (790 mg, 0.99 mmol) in ethyl acetate (7.5 mL) at room temperature, a solution of hydrogen chloride in dioxane (4.0 M, 2.5 mL, 10 mmol) was added. The reaction was stirred for 2 hours and then was diluted with ethyl acetate and washed with aqueous sodium hydroxide, water and brine. The organic layer was dried over magnesium sulfate, filtered, and concentrated to give a 4:1 mixture of **27**:**26** (492 mg) as a yellow oil. In general, this material was used without purification, but a small amount from a previous reaction was purified for analysis by reverse phase HPLC (Symmetry Shield RP18, 7 µm, 30x250mm, 30-80% acetonitrile in water with 0.1% trifluoroacetic acid). Fractions containing the desired material were combined, diluted with water and ethyl acetate, made basic with aqueous sodium hydroxide, and shaken in a separatory funnel. Then, the phases were separated. The organic layer was washed with water (3 times) and brine, dried over magnesium

sulfate, filtered, and concentrated under reduced pressure to give **27** (88 mg) as a colorless oil. NMR (300 MHz, CD_3SOCD_3): δ 7.33 (t, J = 8.4 Hz, 1H), 7.29 (s, 1H), 7.21 (t, J = 8.1 Hz, 1H), 7.06 (dd. J = 8.1, 0.9 Hz, 1H), 7.02 (d, J = 8.4 Hz, 1H), 6.96 (d, J = 8.7 Hz, 1H), 6.82-6.89 (m, 2H), 6.78 (m, 1H), 6.57 (very br. s, 2H), 5.09 (d, J = 14.4 Hz, 1H), 5.03 (d, J = 14.4 Hz, 1H), 5.00 (m, 1H), 3.86-4.08 (m, 6H), 2.68 (m, 2H), 1.83 (d, J = 0.9 Hz, 3H), 1.36-1.56 (m, 4H), 1.19-1.36 (m, 21H).

Compound **28**: Following the general procedure for amide formation, a solution of **27** (475 mg, 0.68 mmol, crude material from previous step, contains approximately 20% **26**) in dimethylformamide (3.4 mL) was reacted with diglycolic anhydride (236 mg, 2.04 mmol) in the presence of N,N-diisopropylethylamine (0.36 mL, 2.04 mmol). Purification was accomplished by automated reverse-phase chromatography (Biotage®, SNAP120 C18, eluting with 30-70% acetonitrile in water). Fractions containing **28** were combined, and the acetonitrile was removed by rotary evaporation. The resulting mixture was extracted with ethyl acetated (twice). The combined organic layers were washed with brine, dried over magnesium sulfate, filtered and concentrated to give **28** (412 mg) as a pale-yellow foam. NMR (300 MHz, CD_3SOCD_3): δ 12.83 (br. s, 1H), 7.87 (br. s, 1H), 7.33 (t, J = 8.3 Hz, 1H), 7.28 (s, 1H), 7.20 (t, J = 8.0 Hz, 1H), 6.98-7.08 (m, 2H), 6.96 (d, J = 7.8 Hz, 1H), 6.82-6.90 (m, 3H), 6.78 (m, 1H), 5.08 (d, J = 14.1 Hz, 1H), 5.02 (d, J = 14.1 Hz, 1H), 4.98 (m, 1H), 4.09 (s, 2H), 3.93 (s, 2H), 3.85-4.08 (m, 6H), 3.06 (q, J = 6.5 Hz, 2H), 2.64 (m, 2H), 1.83 (s, 3H), 1.40-1.60 (m, 2H), 1.13-1.40 (m, 21H).

Compound **29**: Following the general procedure for conjugation of DSPE-PEG-NH_2, **28** (132.8 mg, 0.163 mmol) in dimethylformamide (1.63 mL) was reacted with TSTU (61.1 mg, 0.203 mmol) in the presence of N,N-diisopropylethylamine (0.106 mL, 0.61 mmol) for 2 hours at room temperature. The resulting mixture was added to a solution of DSPE-PEG_{3400}-NH_2 (173

mg, 0.041 mmol) in dimethylformamide (4.1 mL), and the mixture was stirred for 2 days. Purification by size-exclusion chromatography (Sephadex LH20, eluting with methanol) gave **29** (189 mg) as a brown solid.

Compound **30**: Following the general procedure for deprotection of tert-butyl esters, a solution of **29** (178 mg, 0.0356 mmol) in dichloromethane (1 mL) and trifluoroacetic acid (1 mL) was stirred for 2 hours. Purification by size-exclusion chromatography (Sephadex LH20, eluting with methanol) followed by lyophilization from a water/acetonitrile mix gave **30** (171 mg) as an off-white powder. MALDI (Positive mode, dithranol): central mass of distribution: 4729.3.

Compound **31** (Figure S8) was isolated from the 2-step reaction sequence to prepare **30** from **28** (Figure S7), in which intermediate **29** was not purified by size-exclusion chromatography. After acid catalyzed deprotection of the crude conjugate **29**, size-exclusion chromatography (Sephadex LH-20) was conducted, eluting with methanol. The fractions from the second eluting band that was both UV and PMA active were concentrated. The residue was further purified by reverse-phase HPLC (Symmetry Shield RP18, 7 µm, 30x250mm, 30-80% acetonitrile in water with 0.1% trifluoroacetic acid). One fraction was determined to be >90% pure by analytical RP-HPLC, and it was frozen and lyophilized to give **32** as a fluffy white powder. NMR (300 MHz, CD_3SOCD_3): δ 12.23 (br. s, 1H), 7.78 (t, J = 5.9 Hz, 1H), 7.33 (t, J = 8.1 Hz, 1H), 7.27 (s, 1H), 7.20 (t, J = 8.1 Hz, 1H), 6.95-7.08 (m, 3H), 6.85-6.90 (m, 3H), 6.77 (m, 1H), 5.09 (s, 2H), 5.00 (m, 1H), 4.23 (s, 2H), 3.84-4.07 (m, 8H), 3.65 (s, 3H), 3.06 (q, 6.7 Hz, 2H), 2.67 (d, J = 6.9 Hz, 2H), 1.82 (d, J = 0.9 Hz, 3H), 1.15-1.54 (m, 14H).

Cell adhesion assays. Cell adhesion assays were performed as previously described[77]. Wells were coated either directly with substrate (fibronectin, vitronectin, collagen) or with the

appropriate anti-IgG antibody to subsequently capture IgG fusion proteins (VCAM-1-Ig, MAdCAM-1-Ig, ICAM-1-Ig). The concentration of substrate or Ig fusion protein added to the wells was equivalent to the EC_{50} as previously determined by dose-dependent binding curves. The binding buffer was 50 mM Tris-HCl, pH 7.5, 150 mM NaCl (TBS) with 1mM $MnCl_2$ for all assays, except for the αLβ2/ICAM-1 assay in which TBS, 2 mM $MgCl_2$, 5 mM EGTA was used. Mouse integrin adhesion assays were performed identically, except that mouse VCAM-1 was used. For each assay, the cells expressed the appropriate integrin receptor either in recombinant form (K562/α4β1, K562/α4β7, K562/α1β1) or endogenously (Jurkat/α2β1, Jurkat/α4β1, K562/α5β1, HUVEC/αVβ3, HSB/αLβ2, G-361/α9β1, 70Z/3/mouse α4β1). The compounds were dissolved in DMSO to make a 10-mM stock solution. Dilutions were made in binding buffer to yield the desired working concentrations. The concentration of DMSO in the samples was adjusted to equal that of the most concentrated sample before adding to the cells.

Construction of molecular model of α4β1. Modeling was completed on a 16-core 2.4 GHz AMD Opteron system using the 2012 Schrödinger Modeling Suite (Schrödinger LLC). The Prime module was used in the construction of the α4β1 model, which was based on the α4β7 and α5β1 crystal structures[78,79] (entries 3v4v and 3vi4, respectively) as obtained from the Protein Data Bank. The α4 integrin sequence (P13612) and the β_1 integrin sequence (P05556) were obtained from UniProt (http://www.uniprot.org) and downloaded in the FASTA format. The sequence for α4 was truncated to residues 1-587 and read into Prime. Chain A of α4β7 crystal structure was selected as the template for this part of the model. Similarly, the β1 integrin sequence was truncated to residues 4-445, and Chain D in the α5β1 crystal structure was used as the template for that portion of the model. No changes to the initial alignment were necessary

since the sequences were identical to their respective templates. During the build-structure phase, an energy-based build was used for α4 with the 0DU ligand and for β1 with both the calcium and magnesium ions. Heteromultimer modeling was entered, and the α4 and β1 runs were selected. After construction of the raw model, two loops underwent structural refinement: Loop1 (chain A, residues 33-42) and Loop2 (chain B, residues 553-560). These loops were distal from the ligand binding site and not expected to affect the binding region. At this point, we noticed that some bonds in the ligand were incorrect. The bonds were corrected, and the protein portion of the model was prepared. This involved basic protein preparation; assignment of heteroatom states; and H-bond assignment, including PROPKA to assign sidechain ionization. This was followed by Impref "H-only" minimization and then Impref "Minimize All" to root mean square deviation=0.5.

Docking THI0565 into integrin α4β1. The initial geometry optimization of THI565 was performed in PRODRG (Supplementary Fig. S9). Quantum mechanical calculations were completed in FIREFLY with a Pople's 6-N31G* (6 Gaussians) split valence basis set applying Hartree–Fock theory. The derived partial atomic charges were computed from a least-squares fit of the electrostatic potential (ESP) from the Lowdin atomic population and aggregated in charge groups that matched the GROMACS force field. THI565 was docked into the input molecular model of α4β1 by using Autodock Vina 1.1.2 with an unbiased search box (Supplementary Fig. S10) with center (6,40,10)A and size (100,100,100)A and then refined by using a focused box encompassing the putative binding sites, identified from the first round at the interface between the α4 and β1 domain with a center of (2,35,72)A and size of (40,32,40)A. Each trial was assigned an independent random seed. An exhaustiveness of 100 was used with a mode number

of 50. The top 20 poses were selected for each trial. Molecular dynamics simulations were run using GROMACS 5.1.4 with a GROMOS96 43a1 force field. The total system charge was neutralized using the appropriate number of sodium or chloride counterions. An energy minimization was first performed to remove interatomic clashes by using 500 steps of the steepest descent algorithm or until a threshold was met. Next, NVT and NPT energy minimization was done to prepare the complex for equilibration and production MD simulation. A set of 1.0 ns production molecular dynamics simulations was conducted for the protein-compound complex (Supplementary Fig. S11).

Liposome formulation. 1,2-Dihexadecanoyl-sn-glycero-3-phosphocholine (DPPC) and cholesterol were purchased from Lipoid Inc. Diethylenetriaminepentaacetic acid-bis(stearylamide) gadolinium salt (Gd-DTPA-BSA) was purchased from Avanti Polar Lipids. 1,2-Distearoyl-sn-glycero-3-phosphoethanolamine-N-[methoxy(polyethylene glycol)-2000] (DSPE-MPEG$_{2000}$) was purchased from Corden Pharma. Lissamine Rhodamine B 1,2-Dihexadecanoyl-sn-Glycero-3-Phosphoethanolamine, Triethylammonium Salt (rhodamine DHPE) was purchased from ThermoFisher Scientific. All purchased reagents were used without further purification. DSPE-PEG$_{3400}$-THI565 conjugate (referred to as THI0567) was synthesized as described above. DPPC, cholesterol, DSPE-MPEG$_{2000}$, Gd-DTPA-BSA, and DSPE-PEG$_{3400}$-THI567 were respectively constituted on the basis of the desired surface targeting ligand expression at molar proportions shown in Supplementary Table S1. We added rhodamine DHPE (1.0-2.5 mg, 0.2 mol%) to each of the lipid compositions, and particle formulation proceeded as previously described[44]. Briefly, the lipids were dissolved in ethanol (1.0 to 1.2 mL) and then hydrated at ~65°C for 40 minutes in 150 mM saline/10 mM histidine to achieve a lipid

concentration of 50 mM. The mixture was then extruded in a 10-ml Lipex extruder (Northern Lipids Inc.) by using a 400-nm polycarbonate track-etch filter (5 passes) to obtain particles with a mean diameter of ~250 nm. For particles with a mean diameter of ~150 nm, the ensuing formulation was further extruded through a 200-nm polycarbonate filter (8 passes); for particles with a mean diameter of ~100 nm, the formulation was further extruded (5 times) through 100-nm filters. The resulting solution was then dialyzed against 150 mM saline/10 mM histidine. The mean liposome size in the final formulation was determined by dynamic light scattering, and the gadolinium and phospholipid (equivalent phosphorus) concentrations in the formulation were quantified using inductively coupled plasma optical emission spectroscopy (ICP-OES). The number of particles/mL was computed on the basis of the mean particle size and the final lipid concentration in the formulation.

Liposome binding assays. Cells were incubated with indicated concentrations of liposome in binding buffer (50 mM HEPES, pH 7.4, 150 mM NaCl, 2.5 mM KCl, 10 mM $NaHCO_3$, 1 mg/ml glucose) for 1h at room temperature. Background non-specific binding was determined in the presence of EDTA (20 mM). After incubation, cells were washed once in binding buffer and resuspended. Rhodamine B fluorescence was measured on a flow cytometer (LSRII, BD). Binding data are expressed as the geometric mean fluorescence intensity (gMFI). Binding Kds were generated in Prizm Software using the saturation binding equation for "One site - Total and nonspecific binding." Total binding was fit with the equation Y=(Bmax*X/(Kd+X)) + NS*X+BKG; nonspecific binding was fit to the linear equation Y=NS*X+BKG, where X is the particle concentration of liposome, Y is Rhodamine B fluorescence, NS is nonspecific binding, and BKG is background (NS and BKG are shared). In some experiments, after in vitro binding

assays were performed, cells were labelled with the indicated monoclonal antibodies (anti-CD64 mAb or anti-CD3 mAb OKT3) for confocal analysis. Cells were incubated with 10 ug/ml of primary antibody in FACS buffer (PBS, 10% FCS, pH 7.4) for 1h at 4°C. After washing, secondary GAM-FITC (2 ug/ml) was incubated with cells (1h, 4°C), which were then washed, subjected to cytospin onto glass coverslips, air dried, and mounted for confocal imaging.

Fluorescent microscopy. For confocal analysis, all images were obtained by using a Leica TCS SP5 II confocal microscope. The incident laser intensity and image capture settings for each channel were kept constant for all imaging in which control and treatment groups were directly compared. Reconstruction of 3D images was performed by scanning an *XY* plane at multiple *Z* positions and utilizing the 3D-visualization software within the LAS AF software package (Leica). For standard fluorescence, images were captured on a Olympus BX51 fluorescent microscope with Cellsens Dimension imaging software.

References

1. Biediger, R. J. *et al.* Patent No. US6972296 (2005).

2. Koley, D., Colon, O. C., & Savinov, S. N. Chemoselective nitration of phenols with tert-butyl nitrite in solution and on solid support. *Org. Lett.* **11**, 4172-4175 (2009).

3. List, Benjamin *et al.* Practical synthesis of (E)-α,β-unsaturated esters from aldehydes. *Adv. Synth. Catal.* **347**, 1558-1560 (2005).

4. Qiu, X. L. *et al.* Synthesis and biological evaluation of a series of novel inhibitor of Nek2/Hec1 analogues. *J. Med. Chem.* **52**, 1757-1767 (2009).

Supplementary Table S1. Selectivity data of antagonists tested against a panel of integrin targets. Data are presented as the average IC_{50}±SEM from at least 3 experiments.

Integrin	Ligand	IC_{50} (nM) ± SD	
		THI0520	THI0565
α4β1	VCAM-1	0.48 ± 0.07 (n=8)	0.33 ± 0.07 (n=6)
α4β7	MAdCAM-1	90.1 ± 20.2 (n=3)	585 ± 132 (n=3)
α9β1	VCAM-1	1720 ± 310 (n=4)	2480 ± 1335 (n=4)
α1β1	Collagen IV	>10,000 (n=3)	>10,000 (n=3)
α2β1	Collagen I	>10,000 (n=3)	>10,000 (n=3)
α5β1	Fibronectin	>10,000 (n=3)	>10,000 (n=3)
αLβ2	ICAM-1	>10,000 (n=3)	>10,000 (n=3)
αVβ3	Vitronectin	>10,000 (n=3)	>10,000 (n=3)
αIIbβ3	Fibrinogen	>10,000 (n=3)	>10,000 (n=3)

Supplementary Table S2. Constituents of THI0567-targeted liposomal-Gd constructs. DPPC, Cholesterol, DSPE-MPEG$_{2000}$, Gd-DTPA-BSA and DSPE-PEG$_{3400}$-THI567 were respectively constituted based on the desired surface targeting ligand expression at molar proportions shown in the table below.

**Surface ligand THI0567**	**DPPC (mg)**	**Cholesterol (mg)**	**DSPE-MPEG$_{2000}$ (mg)**	**GD-DTPA-BSA (mg)**	**THI0567 (mg)**
0.05%	31.95	40	3	25	0.05
0.25%	31.75	40	3	25	0.25
1.0%	31.0	40	3	25	1.0

Supplementary Table S3. Binding affinities of THI0567-targeted liposomal-Gd against rat, rabbit, and dog cells. Binding assays (using 250 nm, 1.0% targeted ligand) were performed with indicated cells (n=3) as described in the Materials and Methods (Supplement). Curve fitting was performed in with GraphPad Prizm using a 1-site binding model of Total and Non-specific binding. Non-specific binding was determined in the presence of EDTA (20mM).

**Cell line**	**Species**	**Kd (av ± SEM) (pM)**
Jurkat	Human	174.0 ± 21.3[a]
DH82	Dog	111.2 ± 49.1
RH/K34	Rabbit	355.3 ± 37.1
RBL-1	Rat	312.7 ± 53.1

[a]Value is replicated from Figure 2 part D for comparison purposes.

Supplementary Figure S1

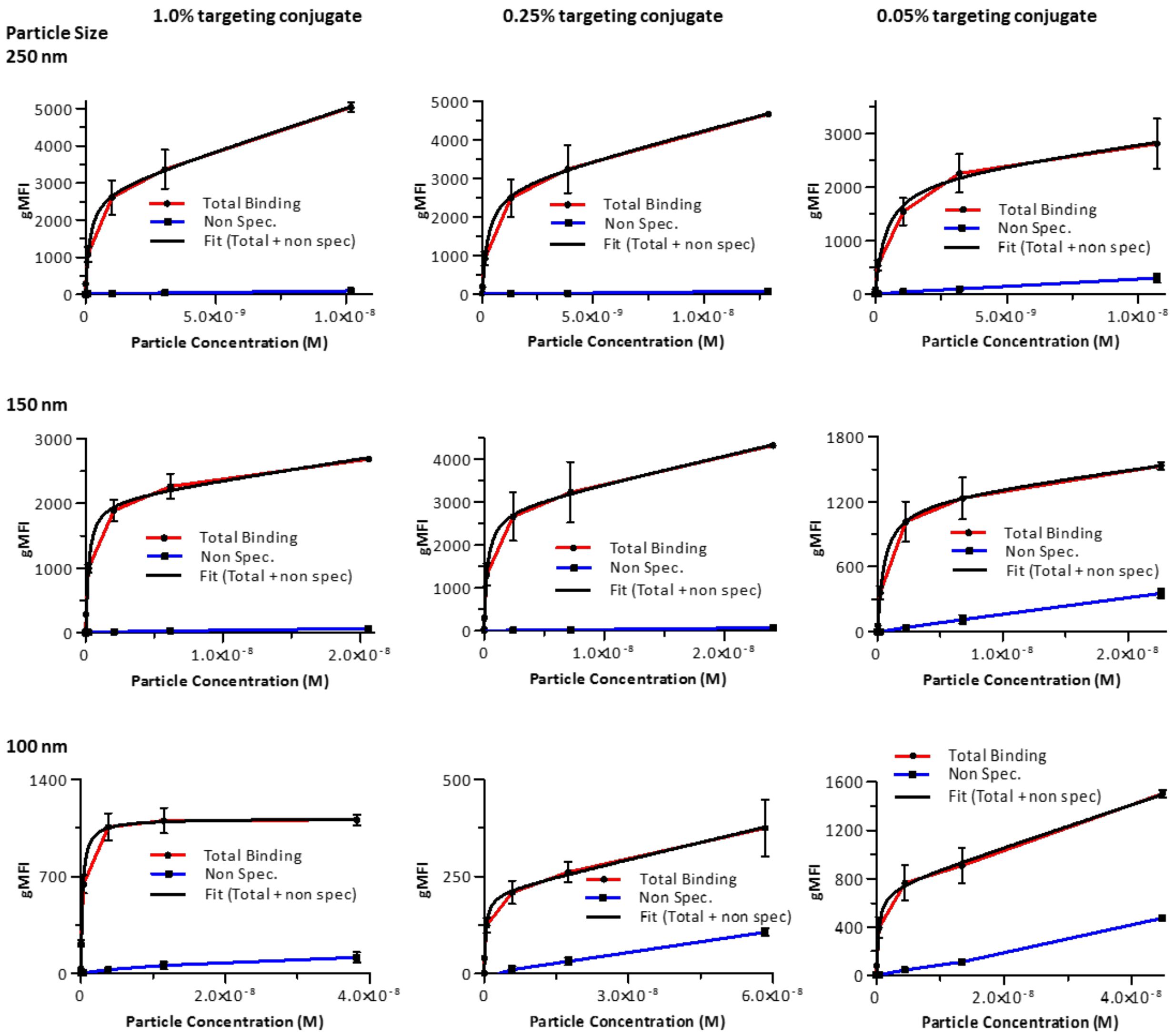

Fig. S1. THI0567-targeted liposome binding assays expressed as the mean gMFI±SEM (n=3). Liposomes ranged in size from 250 nm to 100 nm, with differing levels of targeting agents (1.0%, 0.25%, and 0.05%). Binding assays were performed with Jurkat T cells as described in the Materials and Methods. Curve fitting was performed with GraphPad Prizm using a 1-site binding model of Total and Non-specific binding. Non-specific binding was determined in the presence of EDTA (20mM).

Supplementary Figure S2

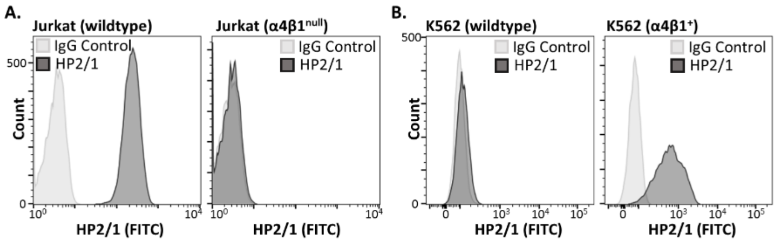

Supplementary Figure S2. Integrin α4β1 expression levels on various cell lines. (A) Jurkat wildtype cells (left panel) and Jurkat $\alpha4\beta^{1null}$ cells (right panel). (B) K562 wildtype cells are shown in the left panel. K562 cells stably expressing the integrin α4β1 are shown in the right panel. For all panels, IgG-control staining is light grey and anti-α4 mAb HP2/1 staining is dark grey.

Supplementary Figure S3

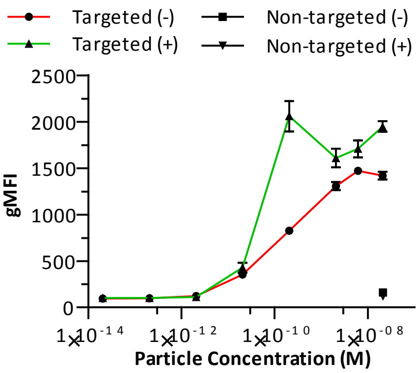

Fig. S3. Binding of targeted liposomes in the presence of serum. Binding of liposome constructs to Jurkat cells in the presence (green line) or absence (red line) of serum (50% v/v). Average gMFI±SEM (n=3 experiments).

Supplementary Figure S4

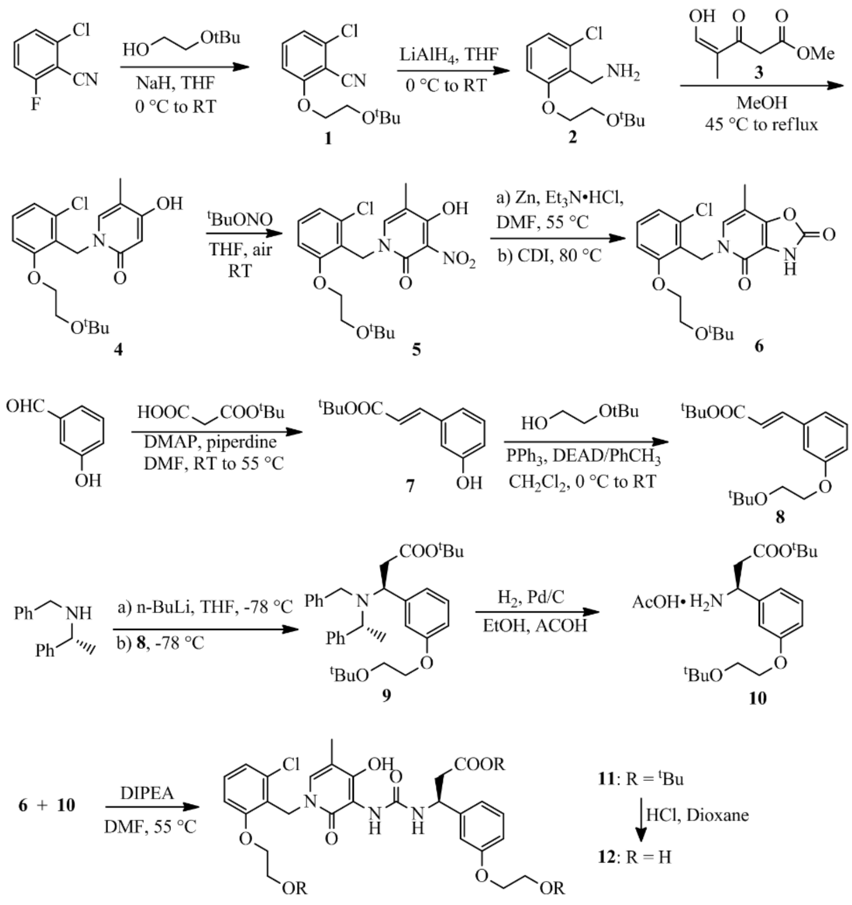

Supplementary Figure S4. Synthetic scheme 1 describing synthesis of compounds 1 through 12.

Supplementary Figure S5

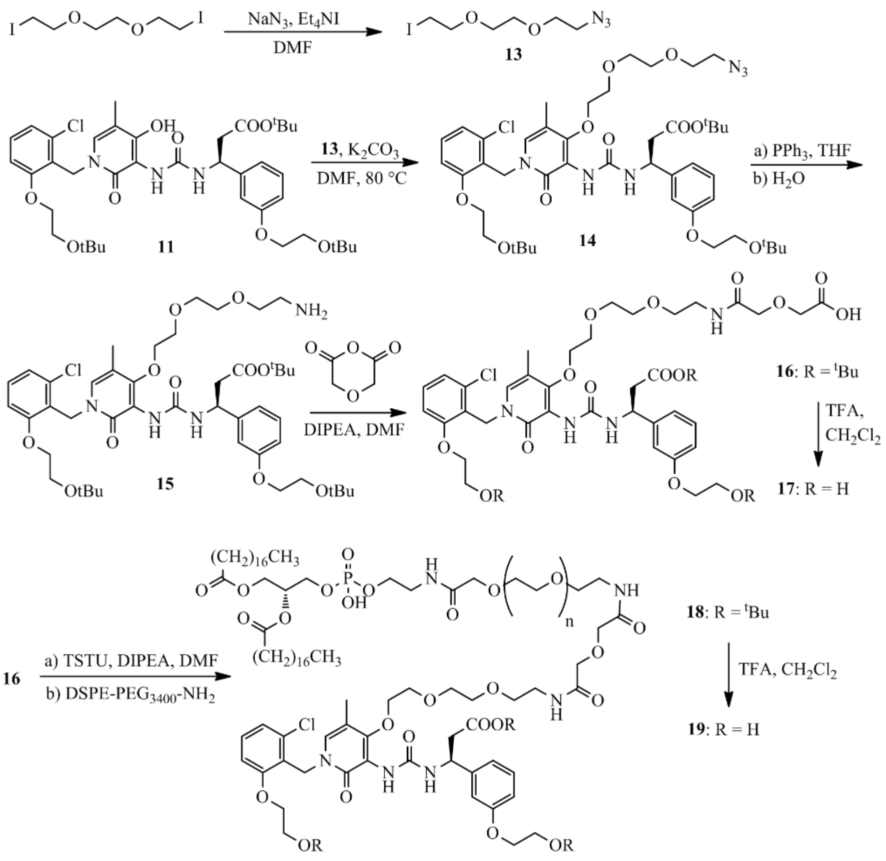

Supplementary Figure S5. Synthetic scheme 2 describing synthesis of compounds 13 through 19.

Supplementary Figure S6

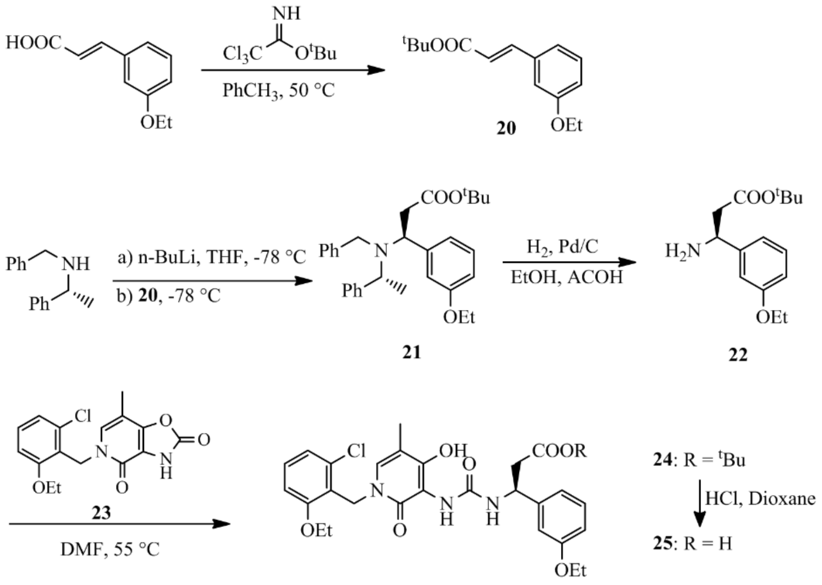

Supplementary Figure S6. Synthetic scheme 3 describing synthesis of compounds 20 through 25.

Supplementary Figure S7

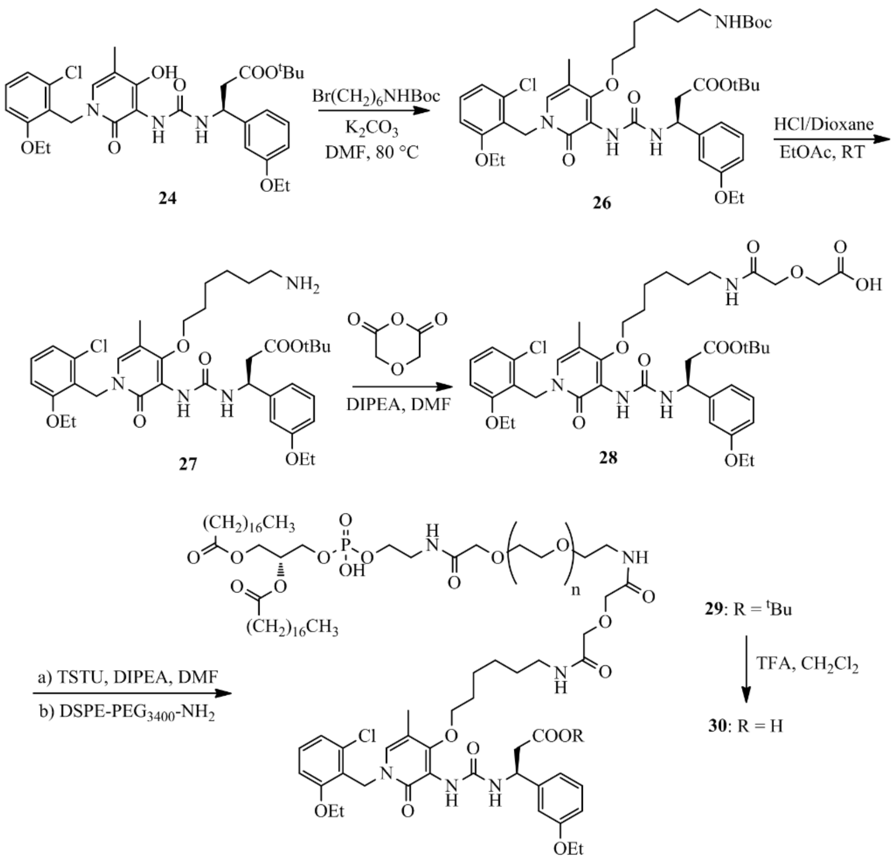

Supplementary Figure S7. Synthetic scheme 4 describing synthesis of compounds 24 through 30.

Supplementary Figure S8

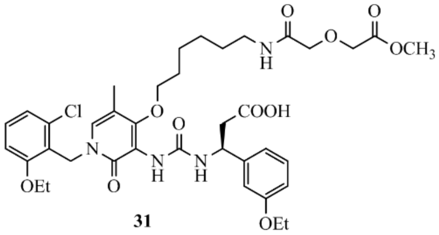

31

Supplementary Figure S8. Structure of compound 31.

Supplementary Figure S9

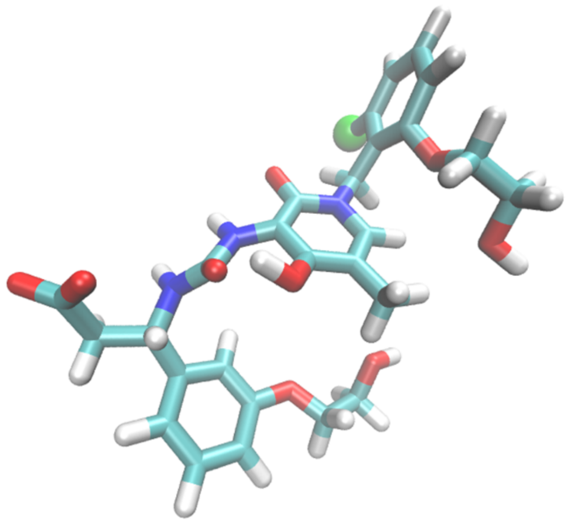

Supplementary Figure S9. THI0565 geometry with a urea in cis and proximal to the pyridone hydroxyl. Geometries were energy minimized and quantum mechanical calculations were evaluated for THI0565 using GAMESS/FIREFLY. Docking was performed with multiple initial conformations that sampled dihedral angles of the urea segment of the molecule in both cis and trans. The remaining rotatable bonds had lower transition energies, allowing robust sampling throughout the docking evaluations in AUTODOCK vina.

Supplementary Figure S10

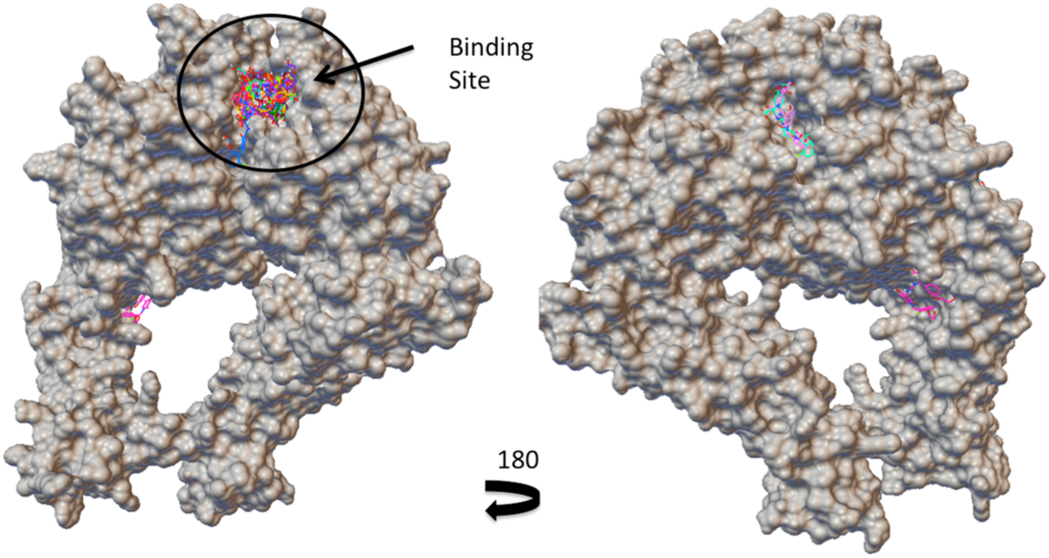

Supplementary Figure S10. Evaluation of docking using a large unbiased search box found that 15 of the top 20 poses clustered in a putative ligand-binding site in the interface between the α4 and β1 subunits of integrin α4β1. A focused box was then evaluated, and the lowest energy pose from the cluster in the putative binding site was chosen for production molecular dynamics simulations using GROMACS to characterize the stable interactions in the protein-compound complex.

Supplementary Figure S11

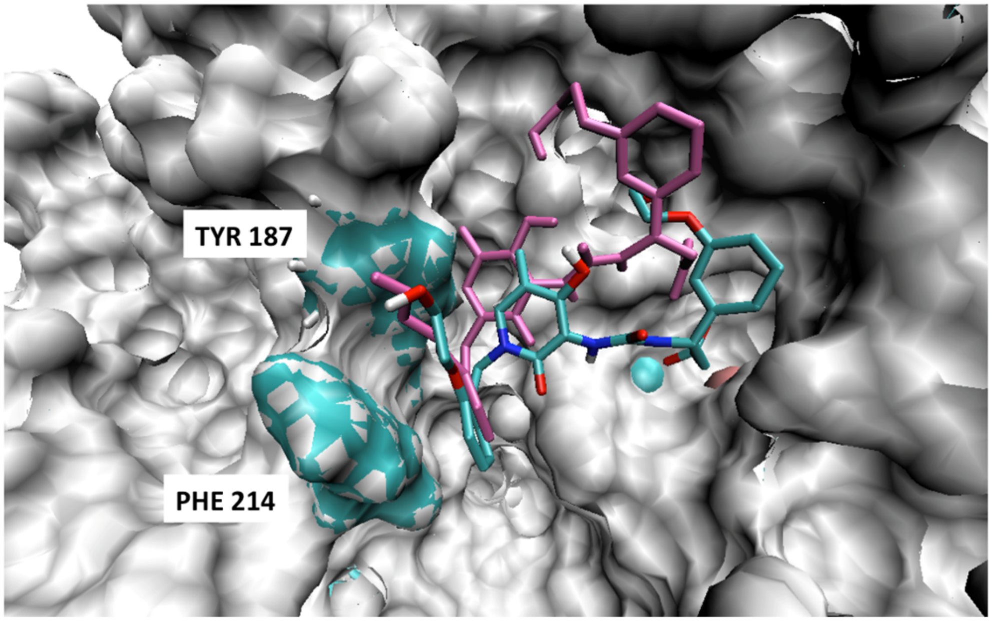

Supplementary Figure S11. The compound THI565 remains stable in the binding pocket over the MD simulation trajectories. The docked result in mauve defined the initial conditions for the production molecular dynamics simulation; the final frame of MD simulation is colored by name and maintains the coordination with the metal ion as the protein breathes. The carboxyl interacts with the Mg ion (pink sphere) with a distance between THI565:OBK/SER227:HG of 2.6 Angstroms. PHE 214 and TYR 187 in cyan anchor the other side of the molecule in the binding site. The pyridone hydroxyl is readily available for conjugating a lipid tail.

Supplementary Movie S1. Internalization of THI0567-targeted liposome. 3D reconstruction of a liposome-loaded Jurkat T cell.

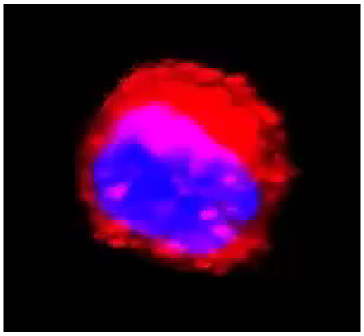

Supplement: Supplementary file 1 — Supplementary Information [file 41598_2018_21893_MOESM1_ESM.pdf]
